# Insights into the GSDMB-mediated cellular lysis and its targeting by IpaH7.8

**DOI:** 10.1038/s41467-022-35725-0

**Published:** 2023-01-04

**Authors:** Hang Yin, Jian Zheng, Qiuqiu He, Xuan Zhang, Xuzichao Li, Yongjian Ma, Xiao Liang, Jiaqi Gao, Benjamin L. Kocsis, Zhuang Li, Xiang Liu, Neal M. Alto, Long Li, Heng Zhang

**Affiliations:** 1 The Province and Ministry Co-sponsored Collaborative Innovation Center for Medical Epigenetics, Key Laboratory of Immune Microenvironment and Disease (Ministry of Education), Haihe Laboratory of Cell Ecosystem, Tianjin Institute of Immunology, Department of Pharmacology, School of Basic Medical Sciences, Tianjin Medical University, 300070 Tianjin, China; 2 State Key Laboratory of Biocatalysis and Enzyme Engineering, School of Life Sciences, Hubei University, 430062 Wuhan, China; 3 Department of Immunology, School of Basic Medical Sciences, Tianjin Medical University, 300070 Tianjin, China; 4 Department of Biochemistry and Molecular Biology, School of Basic Medical Sciences, Tianjin Medical University, 300070 Tianjin, China; 5 Department of Microbiology, University of Texas Southwestern Medical Center, Dallas, TX 75390 USA; 6 State Key Laboratory of Medicinal Chemical Biology, Frontiers Science Center for Cell Responses, College of Life Sciences, Nankai University, 300071 Tianjin, China

**Keywords:** Pathogens, X-ray crystallography, Cell death, Antimicrobial responses

## Abstract

The multifunctional GSDMB protein is an important molecule in human immunity. The pyroptotic and bactericidal activity of GSDMB is a host response to infection by the bacterial pathogen *Shigella flexneri*, which employs the virulence effector IpaH7.8 to ubiquitinate and target GSDMB for proteasome-dependent degradation. Furthermore, IpaH7.8 selectively targets human but not mouse GSDMD, suggesting a non-canonical mechanism of substrate selection. Here, we report the crystal structure of GSDMB in complex with IpaH7.8. Together with biochemical and functional studies, we identify the potential membrane engagement sites of GSDMB, revealing general and unique features of gasdermin proteins in membrane recognition. We further illuminate how IpaH7.8 interacts with GSDMB, and delineate the mechanism by which IpaH7.8 ubiquitinates and suppresses GSDMB. Notably, guided by our structural model, we demonstrate that two residues in the α1-α2 loop make the mouse GSDMD invulnerable to IpaH7.8-mediated degradation. These findings provide insights into the versatile functions of GSDMB, which could open new avenues for therapeutic interventions for diseases, including cancers and bacterial infections.

## Abstract (web summary)

The multifunctional GSDMB protein is an important molecule in human immunity. Here, the authors decipher the molecular mechanisms of GSDMB targeting by the bacterial virulence factor IpaH7.8 and provide insights into GSDMB-mediated pyroptosis.

## Introduction

The gasdermin (GSDM) proteins, mammalian effectors of pyroptosis, play crucial roles in inflammasome-dependent innate immune response^1–5^. The GSDMs, except for GSDMF, adopt a common auto-inhibition conformation with a cytotoxic N-terminal pore-forming domain (NTD) and a C-terminal inhibitory domain (CTD)^5,6^. After proteolytically cleavage of the inter-domain linker by proteases such as caspases and granzymes, NTD would be released and forms oligomeric pore on the cell membrane upon lipid engagement, thereby eliciting target cell death by membrane rupture and leakage^7–10^.

The *GSDMB* gene is present in primates but absent in rodents^11^. GSDMB is a unique member of the GSDM family as it is associated with several different disease classes. GSDMB exhibits distinct functions in its cleaved and uncleaved form, and it recognizes lipids enriched in both mammalian cells and bacterial membranes^12^. Prior to its recognition as a pore-forming cytolysin, genomic and functional studies suggest that GSDMB is likely linked to human disorders, such as asthma, type 1 diabetes, and inflammatory bowel disease^13–18^. It therefore appears that disruption of GSDMB function may result in uncontrolled inflammatory responses. Indeed, a recent study suggests that GSDMB assists in epithelial cell wound healing independently of its pore-forming activity^18^. This work raises the question of whether unmodified GSDMB adopts a similar conformational architecture as other GSDM family members. A second major function of GSDMB is to facilitate tumor clearance in anti-tumor immunity^19^. Unlike most canonical GSDM family members, however, GSDMB is not cleaved and activated by cellular caspases. Rather, serine protease granzyme A (GZMA) delivered from cytotoxic T lymphocytes and natural killer (NK) cells activates GSDMB by site-specific cleavage of the inter-domain linker, therefore liberating NTD and subsequently executing pyroptotic cell death in cancer cells^19^. The CTD of GSDMB adopts a helical-bundle structure^20^, similar to those of GSDMD and GSDMA3^5,6,21,22^. However, the full-length GSDMB, but not other GSDMs, could engage lipids in vitro^20^, suggesting a distinct activation mechanism.

Most recently, this GZMA-mediated activation of GSDMB has been found to restrict bacterial proliferation^23^. Interestingly, GSDMB was shown to directly lyse Gram-negative bacteria. The arms race between hosts and pathogens facilitates their co-evolution. Indeed, the Gram-negative bacterium *Shigella flexneri*, a causative agent of shigellosis, suppresses the GSDMB innate immune defense system by delivering the type III secretion system (T3SS) effector protein IpaH7.8^23^. IpaH7.8 belongs to a family of bacterial ubiquitin E3 ligases featuring an N-terminal variable leucine-rich repeat (LRR) domain, followed by a conserved novel E3 ligase (NEL) catalytic domain at the C-terminus^24^. Upon infection, IpaH7.8 recognizes and ubiquitinates GSDMB, thereby dampening the GSDMB-mediated bacterial lysis through proteasomal degradation of GSDMB^23^. Interestingly, this original report suggests that IpaH7.8 exhibits selective interactions with GSDMB and does not target other GSDM family members. However, IpaH7.8 was recently shown to interact with both GSDMB and GSDMD^25^. This study further demonstrates that GSDMD from mouse is protected from IpaH7.8-mediated ubiquitination through an unknown specificity determinant. These findings are significant in light of the observation that mice also lack GSDMB and are resistant to *Shigella* infection. Thus, it is plausible that IpaH7.8 has shaped the evolution of the GSDM family in rodents. Importantly, however, the molecular mechanism of IpaH7.8-mediated recognition of GSDMB and human GSDMD (hGSDMD) remains poorly understood.

In this work, we report the structure of the GSDMB-IpaH7.8 complex. A combination of structural and biochemical analysis reveals the similarity and diversity of auto-inhibition and pore-forming among GSDMs, as well as the recognition mechanism of IpaH7.8. We further identify the structural elements in GSDMB and GSDMD responsible for the binding preference of IpaH7.8. Together, these findings uncover molecular details in the arms race between *Shigella flexneri* and two human GSDM-family members, which could facilitate the development of therapeutic drugs to treat human disorders and bacterial infectious diseases.

## Results

### Characterization of the GSDMB-IpaH7.8 complex formation

**Fig. 1:**
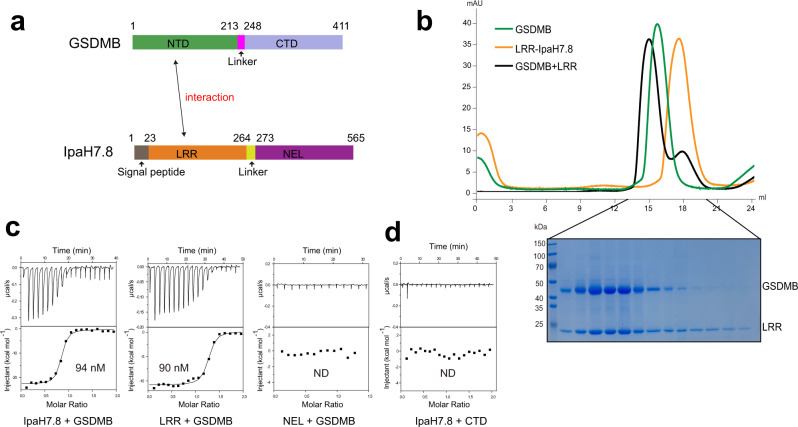
IpaH7.8 binds to GSDMB. **a** Domain organization of GSDMB and IpaH7.8. NTD N-terminal domain, CTD C-terminal domain, LRR leucine-rich repeat domain, NEL novel E3 ligase domain. **b** Superimposed gel-filtration profiles of GSDMB, LRR and GSDMB-LRR complex. Shown on the bottom is SDS-PAGE analysis of the elution fractions corresponding to the GSDMB-LRR complex. **c** ITC-based measurements quantifying the binding affinities between GSDMB and IpaH7.8. ND no detectable binding. **d** ITC analysis of IpaH7.8 binding to CTD of GSDMB. ND no detectable binding. Source data are provided as a Source Data file.

The virulence effector IpaH7.8 has been reported to target GSDMB for degradation^23^. To characterize the interaction between GSDMB and IpaH7.8 (Fig. 1a), we assessed their binding using the size-exclusive chromatography (SEC) assay. The IpaH7.8 protein (residues 23–565) co-migrated with GSDMB in the same fractions (Supplementary Fig. 1a), indicative of a direct interaction between them, which is in agreement with previous studies^23^. Similarly, the N-terminal LRR domain (residues 23–264) of IpaH7.8 was found co-eluted with GSDMB at a stoichiometric ratio (Fig. 1b), whereas the C-terminal NEL domain (residues 265–565) failed to interact with GSDMB (Supplementary Fig. 1b), suggesting that the LRR domain is responsible for the binding of GSDMB. The isothermal titration calorimetry (ITC) measurement further revealed that both IpaH7.8 and LRR domain alone display similar affinities toward GSDMB (Fig. 1c), while no obvious binding was detected for the NEL domain.

Given that GSDMB is supposed to adopt a two-domain architecture, we next mapped the region of GSDMB required for engagement of IpaH7.8. Although we were unable to obtain the NTD due to aggregation and precipitation, the CTD was readily expressed and purified. IpaH7.8 showed no binding to CTD as determined by ITC and SEC (Fig. 1d and Supplementary Fig. 1c), implying that IpaH7.8 recognizes the NTD of GSDMB. Together, our results suggest that the complex formation may be mediated by the interactions between the LRR domain of IpaH7.8 and the NTD of GSDMB.

### Crystal structure of GSDMB-IpaH7.8 complex

**Fig. 2:**
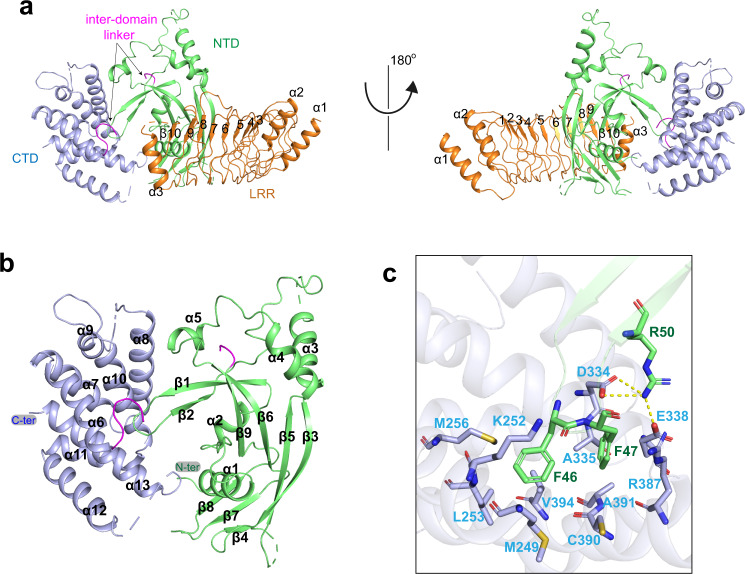
Overall structure of GSDMB in complex with LRR domain of IpaH7.8. **a** Cartoon representation of the GSDMB-LRR structure. The LRR units are indicated by numbers. The color scheme is the same as in Fig. 1a. **b** Overall structure of GSDMB. The NTD and CTD are colored green and light blue, respectively. The inter-domain linker is colored pink. **c** Close-up view of the intramolecular interactions involved in the NTD-CTD interface. Polar interactions are indicated by yellow dashed lines.

We next sought to elucidate the molecular mechanism underlying GSDMB recognition by IpaH7.8. Although crystallization of the GSDMB-IpaH7.8 complex failed after extensive trials, we obtained the crystals of GSDMB in complex with the LRR domain of IpaH7.8, and then determined the complex structure at a resolution of 2.7 Å (Fig. 2a) (Table 1). Almost all the residues of LRR domain except for amino acids 248–254 are clearly visible in the well-defined electron density map. Several loops, including the linker bridging NTD and CTD, are disordered, whereas we observed clear density for the rest of GSDMB.

**Table 1:** X-ray diffraction and refinement statistics

	GSDMB-IpaH7.8 (PDB: 7WJQ)
**Data collection**	
Space group	P 2_1_ 2_1_ 2_1_
Cell dimensions	
*a*, *b*, *c* (Å)	70.471 87.584 102.794
α, β, γ (°)	90 90 90
Resolution (Å)	66.67–2.70 (2.83–2.70)
*R*_sym_ or *R*_merge_	0.077 (0.363)
*I*/σ*I*	22.6 (7.7)
Completeness (%)	97 (95.6)
Redundancy	10.7 (10.8)
**Refinement**	
Resolution (Å)	48.43–2.70
No. reflections	17485
*R*_work_/*R*_free_	0.2102/0.2518
No. atoms	4555
Protein	4534
Ligand/ion	0
Water	0
*B*-factors	53.35
Protein	53.35
Ligand/ion	
Water	52.01
R.m.s. deviations	
Bond lengths (Å)	0.003
Bond angles (°)	0.55

Values in parentheses are for highest resolution shell.

There is one copy of the GSDMB-LRR complex in the crystal asymmetric unit, which is in line with the SEC results that the GSDMB-LRR complex eluted as a heterodimer (Supplementary Fig. 2a). The small-angle X-ray scattering (SAXS) experiments also revealed that the resulting ab initio model and the observed scattering profile of GSDMB-LRR complex agree well with the crystal structure and its theoretical profile, respectively, suggestive of a heterodimer in solution (Supplementary Fig. 2b, c). In addition, both the SEC and analytical ultracentrifugation experiments confirmed that GSDMB and IpaH7.8 form a heterodimer in solution (Supplementary Fig. 2d–f).

### Overall structure of GSDMB

The structure of GSDMB shows a two-domain arrangement in which the NTD connects to the CTD via a flexible loop spanning residues 213–248 (Fig. 2b). The NTD consists of a central bent nine-stranded antiparallel β-sheet, which packs against five α-helices, while the CTD is comprised of a cluster of α-helices. The two domains adopt a side-by-side conformation. The β1-β2 loop is primarily responsible for tethering the NTD to CTD (Fig. 2b). Specifically, the aromatic residues Phe46 and Phe47, the tip of β1-β2 loop, insert into a hydrophobic pocket made up of Met249, Lys252, Leu253, Met256, Ala335, Arg387, Cys390, Ala391 and Val394 (Fig. 2c). The NTD-CTD binding interface is further enforced by polar interactions involving residues Arg50, Asp334 and Glu338. GSDMB adopts an auto-inhibition conformation similar to those of hGSDMD and GSDMA3. GSDMB, hGSDMD and GSDMA3 are auto-inhibited mainly by the β1-β2 loop involved interactions^5,6^, suggesting a general mechanism of auto-inhibition among the GSDM family. However, the binding mode of GSDMB is different from those of GSDMD and GSDMA3. Particularly, the two aromatic residues Phe46 and Phe47 are clamped into a deep hydrophobic pocket, whereas the corresponding aromatic residues of hGSDMD and GSDMA3 fit snugly into two separate hydrophobic pockets, respectively (Supplementary Fig. 3).

### Potential lipid binding sites on GSDMB

**Fig. 3:**
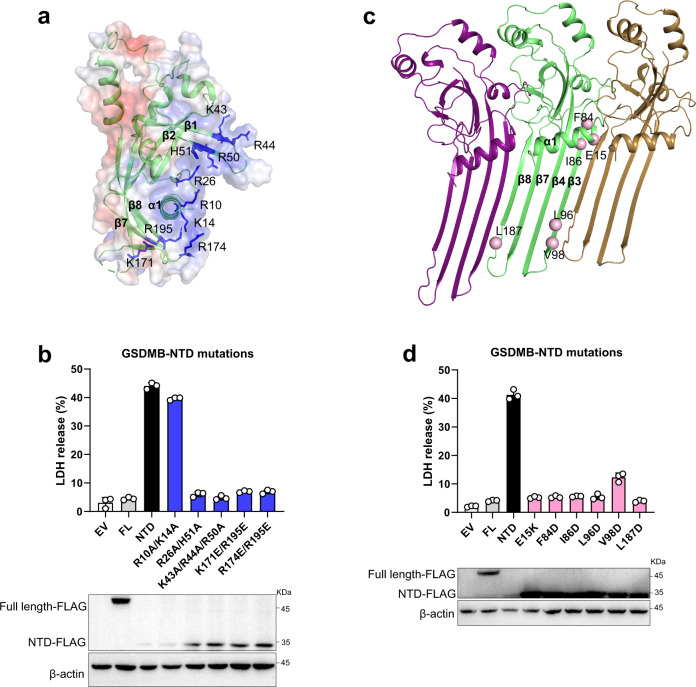
Pore-forming activity of GSDMB. **a** Electrostatic surface representation of NTD-GSDMB. The basic residues are colored in blue and shown in stick representations. **b** Cell death was assessed by LDH cytotoxicity assay in HEK293T cells. Mutations of the basic residues in positively charged regions decreased the NTD-mediated LDH release. All experiments were repeated at least three times (mean ± sd, *n* = 3 independent biological replicates). Bottom, the expression levels of GSDMB proteins. **c** Pore model of NTD-GSDMB. The residues in the oligomerization interface selected for mutation are shown in pink sphere representations. **d** Mutations of the residues in the oligomerization interface reduced the NTD-mediated LDH release. All experiments were repeated at least three times (mean ± sd, *n* = 3 independent biological replicates). Bottom, the expression levels of GSDMB proteins. Source data are provided as a Source Data file.

Several basic patches of NTD in mouse GSDMA3 and hGSDMD, which are covered by the acidic region of CTD in the auto-inhibition state, are reported to interact with the acidic lipid upon pore formation^6,26,27^. Investigation of the GSDMB structure revealed several equivalent positively charged regions (α1, β1-β2 and β7-β8) (Fig. 3a). We then tested the role of these regions in pyroptosis using the lactate dehydrogenase (LDH) cytotoxicity assay in HEK293T cells. Expression of NTD alone led to substantial LDH release (Fig. 3b), indicative of the pore formation and pyroptotic cell death (Supplementary Fig. 4a). Substitutions of the basic residues on β1-β2 (Lys43, Arg44 and Arg50) and β7-β8 (Lys171, Arg174 and Arg195) to alanine or glutamate significantly decreased pyroptosis (Fig. 3b), implying that these two β-sheets may be involved in the lipid recognition as their analogous regions in GSDMA3 and hGSDMD.

The basic residues of α1 helix in GSDMA3 and hGSDMD are presumed to engage the phosphate head of lipid, and mutations of these basic residues impair the pyroptosis^6,26,27^. However, the corresponding mutation (R10A/K14A) in GSDMB had little impact on NTD-mediated pore formation (Fig. 3b), implying the presence of a distinct acidic head recognition site for GSDMB. The NTD of GSDMB has a unique basic patch created by Arg26 and His51 that are acidic or polar residues in other GSDMs (Fig. 3a, Supplementary Figs. 4b and 5). There was a significant reduction in LDH release for double mutation R26A/H51A (Fig. 3b). A similar result was also observed using a cellular assay that reconstitutes the natural mechanism of GSDMB activation by NK cells (Supplementary Fig. 4c, d). Moreover, the R26A/H51A mutation substantially decreased the liposome leakage in the presence of protease compared with WT (Supplementary Fig. 4e). Therefore, it seems that this basic patch is involved in lipid recognition or at least in pore formation. These studies are consistent with the unique lipid binding profile of GSDMB compared to GSDMD and other GSDM family members^23^.

### Potential oligomerization interface

Cryo-EM structures of pores derived from GSDMA3 and hGSDMD demonstrate that substantial conformational arrangement would occur in the NTD domain upon inter-domain cleavage and disruption of the CTD interaction^26,27^. The β3-β5 and β7-β8 sheets, together with their intermediate segments, would transit into new β-hairpins that assemble into the oligomeric pores and integrate into the membrane. However, the globular subdomain comprised of β1-β2, β6-β9 sheets and α-helices is largely maintained. The size of the GSDMB pore (~18 nm) is reported to be comparable to that of the GSDMA3 pore^23,27^, we therefore generated the GSDMB pore model using GSDMA3 as a template.

The oligomerization interface in the globular subdomain is mainly formed by the α1 helix and its surrounding regions, such as the α1-α2 loop and β3 strand (Fig. 3c). An acidic residue in the α1 helix is thought to mediate the oligomerization in GSDMA3 and hGSDMD^6,26,27^. Mutation of the equivalent residue Glu15 in GSDMB dramatically reduced LDH release (Fig. 3d and Supplementary Fig. 4c–e). Another oligomerization interface primarily involves the newly formed β3 and β8 strands (Fig. 3c). Mutations of the residues (Phe84, Ile86, Leu96, Val98 and Leu187) lined in this interface compromised the cell lysis (Fig. 3d and Supplementary Fig. 4c–e), indicating that GSDMB pore formation likely proceeds through a similar conformational rearrangement as other GSDMs family members.

### Interactions between GSDMB and IpaH7.8

**Fig. 4:**
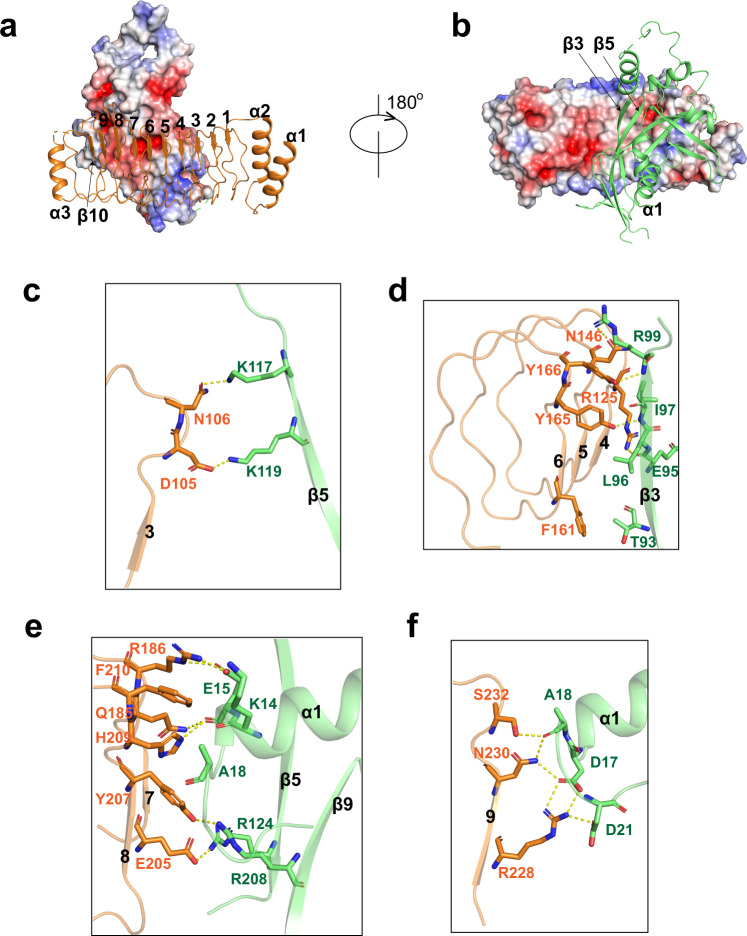
Recognition mechanism of GSDMB by IpaH7.8. **a** Electrostatic surface representations of NTD-GSDMB; LRR is shown in cartoon representation. The LRR units are indicated by numbers. **b** Electrostatic surface representations of LRR domain. NTD-GSDMB is shown in cartoon representation. The secondary elements of GSDMB involved in the binding of IpaH7.8 are labeled. **c–f** Close-up views of interactions in the interface between NTD-GSDMB and LRR domain of IpaH7.8.

The LRR domain of IpaH7.8 possesses nine LRR motifs, assembling into a curved solenoid structure (Fig. 4a). The N-terminal α-helices (α1 and α2), together with the C-terminal α3 helix and β10 strand, mask the tandem LRR units on both ends (Fig. 4a). Although the surface electrostatic potential of the concave face is highly negatively charged, basic and neutral patches are found at the periphery of the LRR domain (Fig. 4b), which are also involved in the binding of GSDMB. The binding affinity between GSDMB and IpaH7.8 was reduced to half in a high salt buffer (Fig. 1c and Supplementary Fig. 6a), suggesting that their association is mediated through a mixture of polar and hydrophobic interactions.

The contact surface of GSDMB recognized by IpaH7.8 is mainly formed by α1 helix, β3 and β5 strands from NTD, whereas no contact was observed between CTD and IpaH7.8 (Fig. 2a). The LRR6-9 segment is primarily responsible for the binding of GSDMB, even though seven of nine LRR units participate in GSDMB engagement. Specifically, LRR3 packs face-to-face against the β5 strand of GSDMB, and their association is maintained by the polar interactions formed between Asp105^LRR3^ and Lys119, and Asn106^LRR3^ and Lys117 (Fig. 4c). The β3 strand of GSDMB is bracketed by LRR4-6 units (Fig. 4d). Arg125^LRR4^ and Asn146^LRR5^ make hydrogen bonds with Glu95 and Arg99 in β3 of GSDMB, respectively. The β3 stand is further stabilized by LRR6-mediated hydrophobic contacts involving residues Phe161^LRR6^, Tyr165^LRR6^ and Tyr166^LRR6^, and Thr93, Leu96 and Ile97 in β3 of GSDMB (Fig. 4d). The C-terminal end of α1 helix in GSDMB is anchored to a basic patch created by LRR7 and LRR8 (Fig. 4e). Arg186^LRR7^ makes electrostatic interactions with Glu15 from α1 of GSDMB, the main chain of which is also hydrogen bonded to Gln185^LRR7^ and His209^LRR8^. The aromatic ring of Phe210^LRR8^ is sandwiched by Lys14 and Ala18, further strengthening α1 helix engagement. LRR8 also engages β5 and β9 strands from GSDMB through the Glu205^LRR8^-Arg124 and Tyr207^LRR8^-Arg208 interactions. A polar cluster of Arg228, Asn230 and Ser232 on LRR9 stabilizes the loop segment (Asp17, Ala18 and Asp21) immediately after the α1 helix (Fig. 4f). The majority of residues involved in the binding of GSDMB are not conserved in other IpaH members, explaining why they are incapable of engaging GSDMB.

### Mutagenesis studies reveal the key residues for GSDMB-IpaH7.8 association

**Fig. 5:**
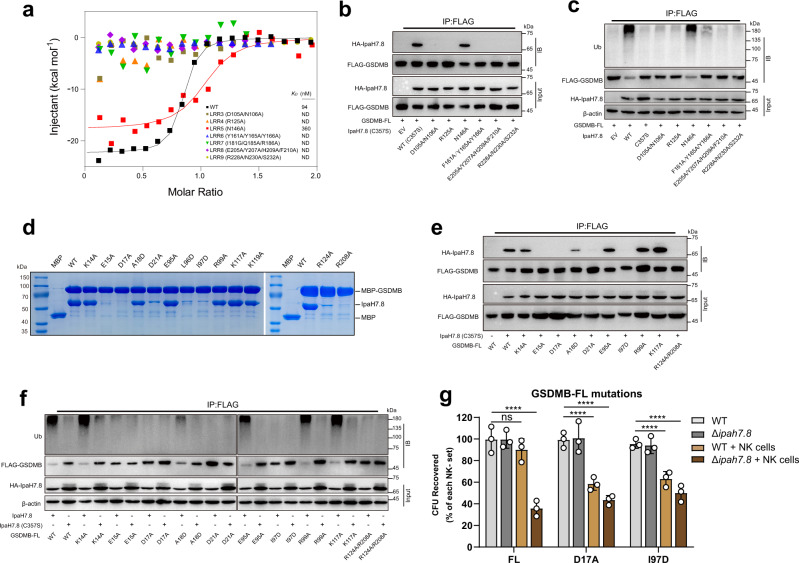
The GSDMB-IpaH7.8 interactions are essential for suppressing NTD-mediated pore formation. **a** Quantification of the binding affinity between GSDMB and IpaH7.8 by ITC. **b** The mutations on IpaH7.8 impair the binding of GSDMB. The protein complex was immunoprecipitated with anti-FLAG antibody, and analyzed by western blotting with the indicated antibodies. **c** Ubiquitination assay for GSDMB by IpaH7.8. Mutations on IpaH7.8 failed to efficiently ubiquitinate GSDMB. Ub, anti-ubiquitin antibodies. **d** In vitro pull-down of IpaH7.8 by MBP-tagged WT or mutant GSDMB proteins. **e** The co-immunoprecipitation assay of IpaH7.8 with WT or mutant GSDMB proteins. **f** Disruption of the GSDMB-IpaH7.8 interactions compromises the ubiquitination of GSDMB. Ub, anti-ubiquitin antibodies. **g** NK cell-mediated killing of intracellular WT or Δ*ipah7.8* *S. flexneri*. The colony-forming units (CFU) of *S. flexneri* recovered from HEK293T cells bearing GSDMB D17A or I97D mutant were significantly reduced when co-cultured with NK cells. All experiments were repeated at least three times. Statistical differences were calculated using Two-way ANOVA (mean ± sd, *n* = 3 independent biological replicates, ns (no significant): *p* = 0.4508, ****p < 0.0001). Source data are provided as a Source Data file.

To validate the importance of the observed GSDMB-IpaH7.8 interactions, we mutated the individual LRR motifs and measured their binding to GSDMB by ITC. All mutations, except for those in LRR5, abolished the GSDMB binding (Fig. 5a). The catalytically inactive IpaH7.8 harboring these mutations also failed to co-immunoprecipitate with GSDMB in HEK293T cells (Fig. 5b). IpaH7.8 also failed to ubiquitinate GSDMB in vitro (Fig. 5c and Supplementary Fig. 7a). Consistent with these assays, loss of function mutations in IpaH7.8 failed to protect GSDMB-expressing HEK293T cells from NK cell-mediated pyroptosis (Supplementary Fig. 6b).

In parallel, mutations were introduced into GSDMB to assess the interaction by in vitro maltose-binding protein (MBP) pull-down assay. IpaH7.8 was efficiently pulled-down by MBP-GSDMB fusion protein, but not by MBP alone (Fig. 5d). The mutations on MBP-GSDMB, including K14A, A18D, E95A, R99A, K117A and K119A, did not or very mildly affected the binding of IpaH7.8; whereas E15A, D17A, D21A, L96D, I97D, R124A and R208A mutants showed significantly decreased binding to IpaH7.8. Importantly, these substitutions abrogated the interaction of GSDMB with IpaH7.8 in HEK293T cells and effectively suppressed the IpaH7.8-mediated ubiquitination (Fig. 5e, f and Supplementary Fig. 7b).

Previous studies have shown that IpaH7.8 protects *Shigella* from NK cell mediated microbicidal activity via GSDMB ubiquitination and degradation^23^. To determine if our structure reflects the native interaction between IpaH7.8 and GSDMB, the viability of *S. flexneri* in cells co-cultured with NK cells was examined. Because IpaH7.8 mutants expressed poorly in *Shigella*, we specifically tested GSDMB mutations (D17A and I97D) that disrupt IpaH7.8 binding, ubiquitination and proteasomal degradation (Fig. 5d–f), but have little effect on NTD-mediated pyroptosis and pore formation (Supplementary Figs. 6c, d). As expected, NK cells failed to inhibit *S. flexneri* growth in cells expressing wild-type GSDMB. In contrast, *S. flexneri* was eliminated by NK cells in the presence of the GSDMB harboring the D17A and I97D mutations that cannot be targeted by IpaH7.8 (Fig. 5g and Supplementary Fig. 7c). These data support the functional significance of GSDMB residues involved in the IpaH7.8 association.

### Interactions between GSDMD and IpaH7.8

**Fig. 6:**
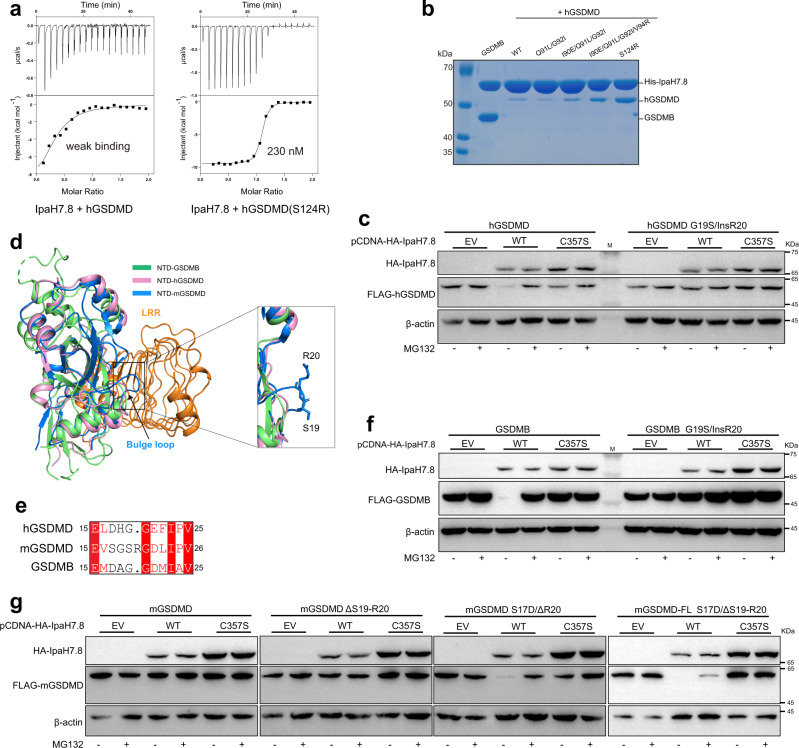
Biochemical and functional characterization of the interactions between GSDMD and IpaH7.8. **a** Quantification of the binding affinity between human GSDMD (hGSDMD) and IpaH7.8 by ITC. **b** In vitro pull-down of WT or mutant hGSDMD proteins by His-tagged IpaH7.8. **c** IpaH7.8 triggered the hGDSMD degradation. Cellular degradation assays in cells expressing the IpaH7.8 and hGSDMD proteins indicated. C357S, IpaH7.8 catalytic dead mutant. insR20, insertion of an additional arginine after the 19th residue. Cells were treated with or without MG132. **d** Structural alignments of NTD-GSDMB with human and mouse NTD-GSDMD. mGSDMD, mouse GSDMD. **e** Sequence alignment of the α1-α2 loop regions across the GSDMs. Invariant residues are shaded red. **f** IpaH7.8 induced the degradation of GDSMB. insR20, insertion of an additional arginine after the 19th residue. Cells were treated with or without MG132. **g** Cellular degradation assays showing that mouse GSDMD (mGSDMD) could be susceptible to IpaH7.8-mediated degradation when mutating both the 17th and 20th residues. Cells were treated with or without MG132. Source data are provided as a Source Data file.

There are conflicting reports on whether IpaH7.8 targets hGSDMD for proteasomal degradation^23,25^. Our ITC and pull-down results showed that IpaH7.8 directly binds to hGSDMD, albeit with a hundreds-fold decreased affinity compared to GSDMB (Fig. 6a, b). Sequence alignment revealed that crucial residues in GSDMB are not conserved in hGSDMD (Supplementary Fig. 5). We next identified the structural determinant of the binding preference by mutating the non-conserved residues in hGSDMD to the equivalent residues in GSDMB. Although most mutations had little impact on the binding of IpaH7.8 in our pull-down assay, the S124R mutation substantially enhanced its interaction with IpaH7.8 (Fig. 6b). ITC results further demonstrated that IpaH7.8 binds to hGSDMD bearing the S124R mutation with a comparable affinity as GSDMB (Fig. 6a). Despite the identification of a single amino acid that contributes to distinct binding affinities between IpaH7.8 and GSDMB or hGSDMD in vitro, hGSDMD was readily co-immunoprecipitated with IpaH7.8 when co-expressed in HEK293T cells (Supplementary Fig. 8a). In addition, expression of IpaH7.8 triggered the degradation of hGSDMD, while the proteasome inhibitor MG132 restored the protein level (Fig. 6c).

Intriguingly, the *GSDMB* gene has been lost in mice and IpaH7.8 could bind but not ubiquitinate mouse GSDMD (mGSDMD)^25^. These data suggest the possibility that IpaH7.8 may have contributed to the evolution of the rodent lineage. Previous studies indicate IpaH7.8 can bind, but not induce proteasomal destruction of mGSDMD^25^. Structural and sequence analysis revealed a unique outward bulge at the corner of the α1-α2 loop, which is mainly formed by Arg20 in mGSDMD (Fig. 6d). This bulge would overlap with the LRR domain of IpaH7.8 in our complex structure, indicating that IpaH7.8 would need to undergo structural rearrangement, such as outward movement, to accommodate mGSDMD. To test the function of this region, an arginine was inserted at the corner of the α1-α2 loop of GSDMB and hGSDMD. Consistent with studies on mGSDMD, IpaH7.8 remained bound to the bulge insertion mutations of GSDMB and hGSDMD but failed to degrade them (Fig. 6c–f and Supplementary Figs. 8a, b). Nonetheless, mGSDMD could not be degraded even when deleting the arginine bulge (Fig. 6e, g and Supplementary Fig. 8c), implying that additional structural divergences may contribute to the IpaH7.8-mediated ubiquitination and degradation. To solve this puzzle, we inspected the amino acid sequence around the arginine bulge. As mentioned above, an acidic residue (Asp17) in the α1-α2 loop of GSDMB is essential for interaction with IpaH7.8. An aspartate residue (Asp17) is also found at the equivalent position in hGSDMD, whereas a serine residue (Ser17) is present in mGSDMD (Fig. 6e). Although either S17D or ΔR20 mutant is resistant to IpaH7.8-mediated degradation (Supplementary Fig. 8d), a combined mutation of S17D and ΔR20 rendered mGSDMD susceptible to IpaH7.8-mediated degradation (Fig. 6g). Thus, these results suggested that the amino acid composition of the α1-α2 loop is the primary determinant for ubiquitination by IpaH7.8.

## Discussion

GSDMB is implicated in many physiological and pathogenic processes, suggesting the versatile nature of GSDMB in primates, thereby making it a promising target for cancer and antimicrobial therapeutics. Here, we solved the crystal structure of GSDMB in complex with IpaH7.8 and characterized the mechanisms underlying auto-inhibition, lipid recognition, and pore formation of GSDMB. We also identified the crucial residues required for GSDMB-IpaH7.8 interaction that supports *S. flexneri* protection against NK-cell mediated lysis. Importantly, these studies help explain controversies about GSDM-family member recognition and provide compelling insights into the mechanism of GSDM protein degradation by IpaH7.8.

Two conserved aromatic residues in the β1-β2 loop may act as the hydrophobic anchor to insert into the membrane for hGSDMD and GSDMA3^5,6,26,27^. Single mutation of either of the two aromatic residues to glycine in hGSDMD almost abolished the LHD release^6^. However, the equivalent F46G or F47G mutation in GSDMB did not change the pore formation activity (Supplementary Fig. 9a). As discussed above, GSDMB also has a unique basic patch around α1 crucial for lipid recognition. Together, it is plausible that GSDMB possesses some different structural features ensuring the membrane engagement. Intriguingly, GSDMB appears to be more discriminating for bacterial membrane^23^, although mammalian membrane can also be integrated by GSDMB. The membrane-binding mode of GSDMB may correlate with the preference. For instance, the unique basic patch might be responsible for interaction with the high amounts of negatively charged phospholipids in the bacterial membrane. Future structural studies of GSDMB pores could provide insights into lipid selectivity.

It has been suggested that the enzymatic activity of the NEL domain is auto-inhibited by LRR domain until substrate binding^28,29^. The structures of LRR domains from IpaH3 and IpaH9.8 are the closest structural homologs of the LRR domain of IpaH7.8^30^. The full-length IpaH7.8 structure predicted by AlphaFold2 with high confidence displays an auto-inhibition conformation, reminiscent of that in IpaH9.8, indicating an apo state of IpaH7.8 (Supplementary Fig. 9b). A hydrophobic pocket at the C-terminus of LRR is thought to engage and lock the NEL domain in IpaH9.8 (Supplementary Fig. 9c). Substrate binding would trigger more flexibility of the LRR C-terminus, thereby probably releasing auto-inhibition by destabilizing the NEL binding^31^. Likewise, an equivalent hydrophobic pocket (Leu216, Val240, Leu244 and Leu247) is also present in the C-terminus of IpaH7.8 LRR (Supplementary Fig. 9d). The aromatic residue in NEL domain of IpaH7.8 is positioned into this hydrophobic pocket, thus possibly anchoring and fixing the NEL domain. It is plausible that auto-inhibition of IpaH7.8 is released in a manner analogous to that of IpaH9.8. Indeed, the LRR C-terminus in the LRR-GSDMB complex adopts a more flexible conformation compared with other regions (Supplementary Fig. 9e). Furthermore, the residues of the hydrophobic pocket in the GSDMB-bound state shifted away from the anchored aromatic residue (Supplementary Fig. 9d), leading to a more open hydrophobic pocket and probably releasing the NEL domain for ubiquitination, consistent with the observation in IpaH9.8-substrate complex^31^.

Structural comparison of LRR-GSDMB with the predicted full-length IpaH7.8 showed that the most divergence occurs in the C-terminal LRR units (Supplementary Fig. 9f). For instance, several residues, including Arg228, Gln257, Tyr259 and Phe260, would undergo a profound rotation and establish contacts with the α1-α2 loop of GSDMB (Fig. 4). These interactions have been demonstrated to be crucial for the ubiquitination of GSDMB by IpaH7.8. Such conformational changes appear to be coupled with the reorganization of the neighboring hydrophobic pocket (Supplementary Fig. 9f). A similar scenario has been described for IpaH9.8. However, it is intriguing to note that an arginine insertion in mGSDMD would clash with the C-terminal LRR units, such as Arg228 and Phe260 (Supplementary Fig. 9g), thereby preventing allosteric activation of IpaH7.8. This analysis may explain why binding to mGSDMD does not trigger ubiquitination by IpaH7.8. Further functional and mutagenesis studies are needed to clarify the activation mechanism.

The LRR domain confers the substrate specificity for the effectors of IpaH family. Previous studies have shown that IpaH7.8, but not other IpaH effectors (such as IpaH3 and IpaH9.8), targets GSDMB for proteasomal degradation^23,25^. It can be explained by the sequence variation of the LRR domains among IpaH family. Furthermore, the divergent substrate binding modes are observed for IpaH family. For instance, human guanylate-binding proteins are reported to bind the N-terminal region of the LRR domain of IpaH9.8^31,32^. In contrast, the IpaH family effector SspH1 target human PKN1 mainly via the C-terminal part of the LRR domain^33^, reminiscent of the IpaH7.8-GSDMB association. Notably, IpaH7.8 predominantly recognizes the β-strands structural elements of GSDMB, while SspH1 binds the coiled-coil subdomain of PKN1 with a relatively smaller interface. It seems that the shape and electrostatic surfaces also dictate the substrate specificity for IpaH proteins and their targets.

Interestingly, almost all the residues on GSDMB essential for interacting with IpaH7.8 are also crucial for pore formation. Presumably, the *Shigella* pathogen has evolved a delicate strategy to suppress host immunity besides protein degradation, in which the N-terminal LRR domain alone may also inhibit the pore-formation (Supplementary Fig. 9h). In such a scenario, the *Shigella* species harboring the naturally occurring incomplete NEL domain would exert the inhibition effects against membrane permeabilization as well.

In addition to targeting GSDMB, IpaH7.8 was found to ubiquitinate the primary executioner of Caspase-1/4/5 mediated pyroptosis, GSDMD^25^. This finding was somewhat surprising since *S. flexneri* has been shown to induce Caspase-1 dependent cell death in both murine and human macrophage^34,35^. We found that the binding affinity of IpaH7.8 to hGSDMD was hundreds-fold lower than GSDMB in vitro. This difference in affinity likely explains why GSDMD was not found as a substantial target in other studies performed in vitro^23^. Indeed, amino acid substitutions in GSDMD that mimic GSDMB restore the strong interaction with IpaH7.8. Despite these in vitro observations, however, we found that hGSDMD was targeted for proteasome-mediated destruction in cells. These data suggest that additional cellular factors may be required to facilitate the GSDMD-IpaH7.8 association required for ubiquitination. Such a model would explain why certain cell types (e.g., human macrophage) undergo GSDMD-mediated pyroptosis in response to *S. flexneri*, whereas others do not (e.g., epithelial cells).

Further analysis of the interaction between IpaH7.8 and murine GSDMD indicates that additional layers of regulation are embedded in the GSDM protein recognition mechanism. Interestingly, the first identified substrate for IpaH7.8 was murine NLRP1 and it was shown that IpaH7.8 ubiquitination of NLRP1 induces mGSDMD-mediated pyroptosis in macrophage^36^. In agreement with this finding, mGSDMD has evolved a mechanism to avoid IpaH7.8-mediated ubiquitination^25^. Our structural and functional analysis further resolves this host defense system. We found that a bulge created by Arg20 at the corner of the α1-α2 loop in mGSDMD prevented IpaH7.8-mediated proteasomal destruction. Remarkably, introduction of an equivalent bulge at the α1-α2 loop in GSDMB and hGSDMD also prevented IpaH7.8-mediated degradation. These data provide a structural rationale for the species selectivity of IpaH7.8 and explain how murine inflammasomes induce macrophage pyroptosis during *S. flexneri* infection^34^. As discussed in more detail above, these findings may also help elucidate the substrate activation mechanism of the NEL family of effector proteins.

In summary, we deciphered the molecular mechanisms of GSDMB targeting by the bacterial virulence factor IpaH7.8 as well as GSDMB-mediated pyroptosis (Supplementary Fig. 9i). Once *S. flexneri* infects host cells, T3SS delivers virulence factors to interfere with host defense responses to create an immune tolerant environment for survival advantages. One of the most conserved host defense mechanisms is GSDM-mediated pyroptosis, which activates both innate and adaptive arms of the immune system to clear pathogens. However, *S. flexneri* evolves an E3 ligase IpaH7.8 to disrupt the cell death by targeting the pyroptosis effector GSDMB/GSDMD for ubiquitination and degradation, which diverts the infected cells from hostile to a friendly environment for the bacteria. Furthermore, the degradation of GSDMB/GSDMD saves *S. flexneri* from a fate of lysis. In this report, we determined the crystal structure of GSDMB-IpaH7.8 complex, and unveiled key residues for their association, which would provide structural guidance for anti-microbial drug design. Future research and drug development targeting the NTD-CTD interface would be of clinical importance for cancers, auto-immune diseases, and microbial infection.

## Methods

### Protein expression and purification

The fragment encoding IpaH7.8 was synthesized and inserted into pET-28-MHL with an N-terminal His tag. The coding sequences of GSDMB (isoform 1, UniProtKB: Q8TAX9) and hGSDMD (isoform 1, UniProtKB: P57764) was cloned into pET-His-MBP with an N-terminal His-MBP tag followed by a TEV protease cleavage site. The resultant plasmids were transformed into *Escherichia coli* BL21 (DE3) cells. Cells were cultured at 37 °C in the LB medium supplemented with antibiotics. When the OD 600 reached 0.6, protein expression was induced by adding 0.2 mM isopropyl β-D-thiogalactopyranoside (IPTG) at 16 °C.

Cells were harvested and resuspended in lysis buffer (25 mM Tris-HCl pH 8.0, 300 mM NaCl, 5 mM imidazole, 2 mM β-mercaptoethanol), and lysed by sonication on ice. Cell lysates were cleared by centrifugation at 13,600 *g*, and isolated supernatant was incubated with Ni-NTA resin (QIAGEN). After extensive wash, the target protein was eluted with lysis buffer supplemented with 300 mM imidazole. TEV was added to the eluates to remove His or His-MBP tag. Further purification of target protein was performed by anion-exchange chromatography (HiTrap Q, Cytiva), followed by size-exclusion chromatography (Superdex 200 increase 10/300, Cytiva) in a buffer containing 25 mM Tris-HCl pH 8.0, 150 mM NaCl, 2 mM DTT.

All mutations were obtained by QuikChange site-directed mutagenesis method. To obtain the GSDMB-IpaH7.8 complex, the two plasmids were co-transformed into competent cells. These mutant proteins, GSDMB-IpaH7.8 complex, and GSDMD proteins were expressed and purified using the same protocol as above.

### Crystallization, data collection and structure determination

GSDMB (residues 1-411)-IpaH7.8 (residues 23–264) protein complex was concentrated to 14 mg/ml for crystallization trials. Stick-shaped crystals grew at 18 °C using the sitting-drop vapor diffusion method by mixing l µl protein and l µl reservoir solution containing 200 mM NaCl, 100 mM HEPES-sodium pH 7.2, 12% PEG 8000, 50 mM ammonium sulfate, 3% (w/v) 1, 6-hexanediol. The crystals were cryo-protected in mother liquor supplemented with 20% glycerol and flash-frozen in liquid nitrogen for data collection.

X-ray diffraction data were collected at beamline BL18U1 of Shanghai Synchrotron Radiation Facility (SSRF) using a Pilatus3 6 M detector^37^. All diffraction data sets were automatically processed using autoPROC^38^. The complex structure was solved by molecular replacement with Phaser in the CCP4i2 package^39^, and the CTD of GSDMB (PDB:5TJ4) and the LRR domain of IpaH7.8 generated by AlphaFold2^40,41^ were used as search models. Automated model building was performed by Buccaneer of CCP4i2 package^42^. Improvement of the initial model was carried out manually by Coot^43^, and the refinement was conducted using REFMAC5^44^ and phenix.refine^45,46^.

### Isothermal titration calorimetry

All the protein samples for ITC were prepared in 25 mM Tris-HCl pH 8.0, 150 mM NaCl unless otherwise stated. The ITC titrations were carried out using the MicroCal PEAQ-ITC system (Malvern) at 25 °C. Each titration was conducted by injecting IpaH7.8 proteins into the cell containing GSDMB or GSDMD proteins. The binding data were analyzed using the MicroCal PEAQ-ITC software. The ITC experiments were repeated at least two times.

### Size-exclusion chromatography assay

To determine the interaction between GSDMB and IpaH7.8, the gel-filtration assay was carried out using Superdex 200 Increase 10/300 GL column (Cytiva). Purified proteins were mixed at a 3:1 molar ratio on ice for 1 h. The mixture was then subjected to a pre-equilibrated column in buffer containing 25 mM Tris-HCl pH 8.0, 150 mM NaCl, 2 mM DTT. Peak fraction samples were separated by SDS-PAGE and visualized by Coomassie blue staining.

### MBP pull-down assay

MBP-tagged GSDMB proteins (~100 µg) were incubated with amylose resin in binding buffer (25 mM Tris-HCl pH 8.0, 200 mM NaCl, 3 mM β-mercaptoethanol) at 4 °C for 1 h. Tag-free IpaH7.8 proteins (~400 µg) were then added and incubated for another 1 h. The resin was extensively washed with binding buffer. Bound proteins were eluted with the binding buffer supplemented with 25 mM maltose. Protein samples were analyzed by SDS-PAGE.

### His pull-down assay

His-tagged IpaH7.8 proteins (~100 µg) were incubated with Ni-NTA resin in binding buffer (25 mM Tris-HCl pH 8.0, 200 mM NaCl, 3 mM β-mercaptoethanol) at 4 °C for 1 h. Tag-free GSDMB and hGSDMD proteins (~400 µg) were then added and incubated for another 1 h. The resin was washed with the binding buffer supplemented with 10 mM imidazole. Bound proteins were eluted with the binding buffer supplemented with 500 mM imidazole. Protein samples were analyzed by SDS-PAGE.

### Small-angle X-ray scattering

All the protein samples were prepared in a buffer containing 25 mM Tris-HCl pH 8.0, 150 mM NaCl. Synchrotron Small-angle X-ray scattering (SAXS) data were collected at beamline BL19U2 of SSRF using a Pilatus 2 M detector (DECTRIS, Switzerland). The scattering was recorded in the range of the momentum transfer 0.010 Å^−1^ < s < 0.450 Å^−1^, where s = 4πsinθ/λ, 2θ is the scattering angle, and λ = 1.033 Å is the X-ray wavelength. The solutions were loaded in a flow-through quartz capillary cell with a diameter of 1.5 mm and a wall thickness of 10 µm, temperature controlled at 23 °C. The radiation damage was checked with 20 successive exposures of 1.0 s.

All the collected two-dimensional images were processed and converted to one-dimensional intensity curves by BioXTAS RAW^47^. Further data processing and analysis were performed by the PRIMUS software^48^. Distance distribution and molecular weight were calculated using GNOM^49^, and ab initio modeling was carried out by DAMMIF^50^ to reconstruct the low-resolution 3D structure. The fit quality between model and experimental SAXS profile was evaluated by CRYSOL and SREFLEX of ATSAS package^51^.

### Analytical ultracentrifugation

Purified proteins were diluted to ~1 mg/ml in a buffer containing 25 mM Tris-HCl pH 8.0, 150 mM NaCl. Sedimentation velocity measurements of IpaH7.8 and GSDMB-IpaH7.8 complex were carried out by a Beckman optima XL-I analytical ultracentrifugation. The sedimentation coefficient was analyzed by SEDFIT and SEDPHAT programs^52^.

### Cell culture and immunoprecipitation assays

For the co-immunoprecipitation (IP) assay of GSDMB/GSDMD and IpaH7.8, HEK293T cells were cultured in Dulbecco’s Modified Eagle Medium (DMEM) complemented with 10% fetal bovine serum (FBS). IpaH7.8 was cloned into the pcDNA3.1 vector, and GSDMB or GSDMD was inserted into the pLV-IRES-eGFP vector. The resultant plasmids coding for HA-IpaH7.8 were transfected with FLAG-GSDMB (C-terminal FLAG) or FLAG-GSDMD (C-terminal FLAG) into HEK293T cells using the Lipo8000 transfection reagent (Beyotime). After 24 h culture, transfected cells were lysed in IP lysis buffer (50 mM Tris-HCl pH 8.0, 150 mM NaCl, 1% NP40) supplemented with protease inhibitor cocktail (Roche) and PMSF. FLAG-beads were added to the cell lysate and incubated overnight at 4 °C. Beads were washed with IP lysis buffer, and target proteins were eluted by boiling with SDS-PAGE loading buffer. Protein samples were loaded onto SDS-PAGE gel and continuously transferred to PVDF membranes. The PVDF membranes were blocked in 5% BSA TBS-T buffer for 1 h and incubated with primary anti-FLAG antibody (CST) or anti-HA antibody at 4 °C overnight. PVDF membranes were then washed with TBS-T buffer before incubated with secondary HRP-linked anti-rabbit antibody (CST) at room temperature for 1 h. Visualization of protein bands was performed by incubating chemiluminescence reagent with the Tanon system (Tanon).

For the study of GSDMB ubiquitination by IpaH7.8, HEK293T cells were co-transfected with HA-IpaH7.8 and GSDMB-FLAG plasmids. Transfected cells were cultured for 24 h, then treated with 10 μM MG132 for 2 h. Cells were washed two times with PBS and lysed in IP lysis buffer. Productions of ubiquitination in cell lysates were detected via co-IP and western bolt as above. To test the effect of IpaH7.8 in substrates stability, HEK293T cells were co-transfected with indicated expression plasmids encoding GSDM substrates, and IpaH7.8 plasmids. At 16 h post-transfection, 0.5–1 μM MG132 (MedChemExpress, HY-13259) was added and treated for 12–24 h. Cells were lysed with RIPA (Millipore). Western blot was used to detect the protein level.

### Cytotoxicity assay

To determine the ability of NTD-GSDMB or NK cells-mediated GSDMB-dependent pyroptosis, a LDH activity was used as the readout of HEK293T lytic cell death. CytoTox 96 Non-radioactive Cytotoxicity Assay kit (Promega) was used for the measurement of LDH activities in the cell culture medium. All the experiments were performed according to the manufacturer’s instructions. The amount of released LDH was presented as a percentage of the total LDH content.

### Liposome dye release assay

Liposome dye release assay was modified from a previous study^53^. The *E. coli* polar extraction was hydrated at 5 mg/ml with 50 mM HEPES, 150 mM NaCl (lipid buffer) plus 10 mM 5(6)-carboxyfluorescein (CF) and gently vortexed till the lipid was distributed evenly into the buffer. Lipid suspension was extruded 31 times through a 100 nm polycarbonate membrane. Liposome containing CF was purified by Sephadex G-25 desalt columns and diluted to lipid buffer at 300 μM. Baseline reading of untreated liposome containing CF as Ft0 was measured by excitation at 490 nm and emission at 520 nm for 2 min containing 6 cycles. WT GSDMB and its mutations were added to the reactions at 5 μg in a final volume of 100 μl and the Ftn value of liposome released fluorescence was continuously monitored for another 23 min. Then, 10 μl 1% Triton X-100 was added to each well for an additional 2 min to obtain the max fluorescence mean value Ftm. The percentage of each cycle CF release was calculated with the formula %(Ftn-Ft0)/(Ftm-Ft0).

### NK cell-mediated killing and intracellular bacterial killing assay

NK92MI cells were expanded in NK cell medium (alpha Minimum Essential Medium, α-MEM) complemented with 20% FBS, 0.2 mM inositol, 0.1 mM β-mercaptoethanol, 0.02 mM folic acid, 10 mM HEPES and non-essential amino acid. WT or mutant GSDMB stably overexpressing HEK293T cells were plated at 1 × 10^4^ cells per well. Then, NK92MI cells were added into each well at a ratio of 8:1 to HEK293T cells and co-cultured in the NK cell medium. After 6 h, supernatant was collected to measure the LDH release. At the same time, the baseline or maximum LDH activities were acquired from each WT or mutant GSDMB stably overexpressing HEK293T cells cultured alone in NK cell medium for 6 h.

WT or Δ*ipah7.8* mutant *Shigella flexneri* M90T was recovered in LB overnight and diluted the next day with fresh LB at 1:50 for another 3 h culture. The bacterium was prepared by centrifugation at 4000 × *g* for 5 min and resuspended with DMEM/HEPES. HEK293T cells stably expressing WT or mutated GSDMB were plated at 5 × 10^4^ per well in polylysine precoated 48-well plates. Cells were infected with *S. flexneri* at MOI 1:100. After 1.5 h incubation, cells were washed with DMEM/HEPES three times and cultured in the complete medium containing 50 μg/ml gentamicin for another 1 h. Then the medium was changed to NK cell medium without gentamicin and NK92MI cells were added to the infected HEK293T cells at a ratio of 10:1. After co-cultured for 3 h, cells were washed with PBS and lysed with 1% Triton X-100. Cell lysates were diluted serially and plated on LB agar overnight. Bacterial colonies were counted, and the CFU recovery was calculated as the percentage of bacterial colonies recovered from the infected HEK293T cells co-cultured with NK cells compared to that of the infected HEK293T cells.

To examine the NK cells-induced cleavage of GSDMB mutations, NK killing assay or intracellular bacterial killing assay was performed in 12-well plates and the total protein was extracted to detect the cleaved GSDMB by Western blot.

### Cellular protein degradation assays

The IpaH7.8, GSDMB and GSDMD plasmids were constructed as described above. pcDNA3.1-HA-IpaH7.8 and pLV-GSDMs-FLAG were co-transfected into HEK293T at a molar ratio of pcDNA3.1:pLV = 2:1. After transfecting for 16 h, MG132 or equal volume of DMSO was added into MG132 + or MG132− well. For detecting the degradation of GSDMB, the cells were cultured with MG132 of 1 μM for 12 h. For detecting the degradation of human or mGSDMD, the cells were cultured with MG132 of 0.5 μM for 24 h. Then, the cells were lysed with RIPA (Millipore). Western blot was used to detect the protein levels.

### Statistics and reproducibility

All the statistical data were drawn and analyzed with Graphpad Prism8. One-way or Two-way ANOVA was adopted to determine the differences as legend described. *P* values were shown by ns (no significant) or star (*p < 0.05, **p < 0.01, ***p < 0.001, ****p < 0.0001). All figures are representative of at least three experiments unless otherwise noted.

### Reporting summary

Further information on research design is available in the Nature Portfolio Reporting Summary linked to this article.

## Supplementary information

Supplementary Information

Reporting Summary

## Data Availability

The structure factors and atomic coordinates for GSDMB in complex with IpaH7.8 have been deposited to the Protein Data Bank with accession number of 7WJQ. Source data are provided with this paper.

## Source data

Source Data
