# Supplementary material for: Insights into the GSDMB-mediated cellular lysis and its targeting by IpaH7.8
Source: Nat Commun. 2023 Jan 4;14:61. doi: 10.1038/s41467-022-35725-0 (PMC9813358; doi:10.1038/s41467-022-35725-0)

Supplementary information

Insights into the GSDMB-mediated cellular lysis and its targeting by IpaH7.8

Hang Yin[1,2#], Jian Zheng[1,3#], Qiuqiu He[1,4#], Xuan Zhang[3], Xuzichao Li[4], Yongjian Ma[4], Xiao Liang[4], Jiaqi Gao[4], Benjamin L. Kocsis[5], Zhuang Li[2], Xiang Liu[6], Neal M. Alto[5], Long Li[1,3]*, Heng Zhang[1,4]*

1. The Province and Ministry Co-sponsored Collaborative Innovation Center for Medical Epigenetics, Key Laboratory of Immune Microenvironment and Disease (Ministry of Education), Haihe Laboratory of Cell Ecosystem, Tianjin Institute of Immunology, Department of Pharmacology, School of Basic Medical Sciences, Tianjin Medical University, Tianjin 300070, China
2. State Key Laboratory of Biocatalysis and Enzyme Engineering, School of Life Sciences, Hubei University, Wuhan 430062, China
3. Department of Immunology, School of Basic Medical Sciences, Tianjin Medical University, Tianjin 300070, China
4. Department of Biochemistry and Molecular Biology, School of Basic Medical Sciences, Tianjin Medical University, Tianjin 300070, China
5. Department of Microbiology, University of Texas Southwestern Medical Center, Dallas, TX 75390, USA
6. State Key Laboratory of Medicinal Chemical Biology, Frontiers Science Center for Cell Responses, College of Life Sciences, Nankai University, Tianjin 300071, China

#These authors contributed equally: Hang Yin, Jian Zheng and Qiuqiu He

*Correspondence: Long.Li@tmu.edu.cn (L.L.), zhangheng134@gmail.com (H.Z.)

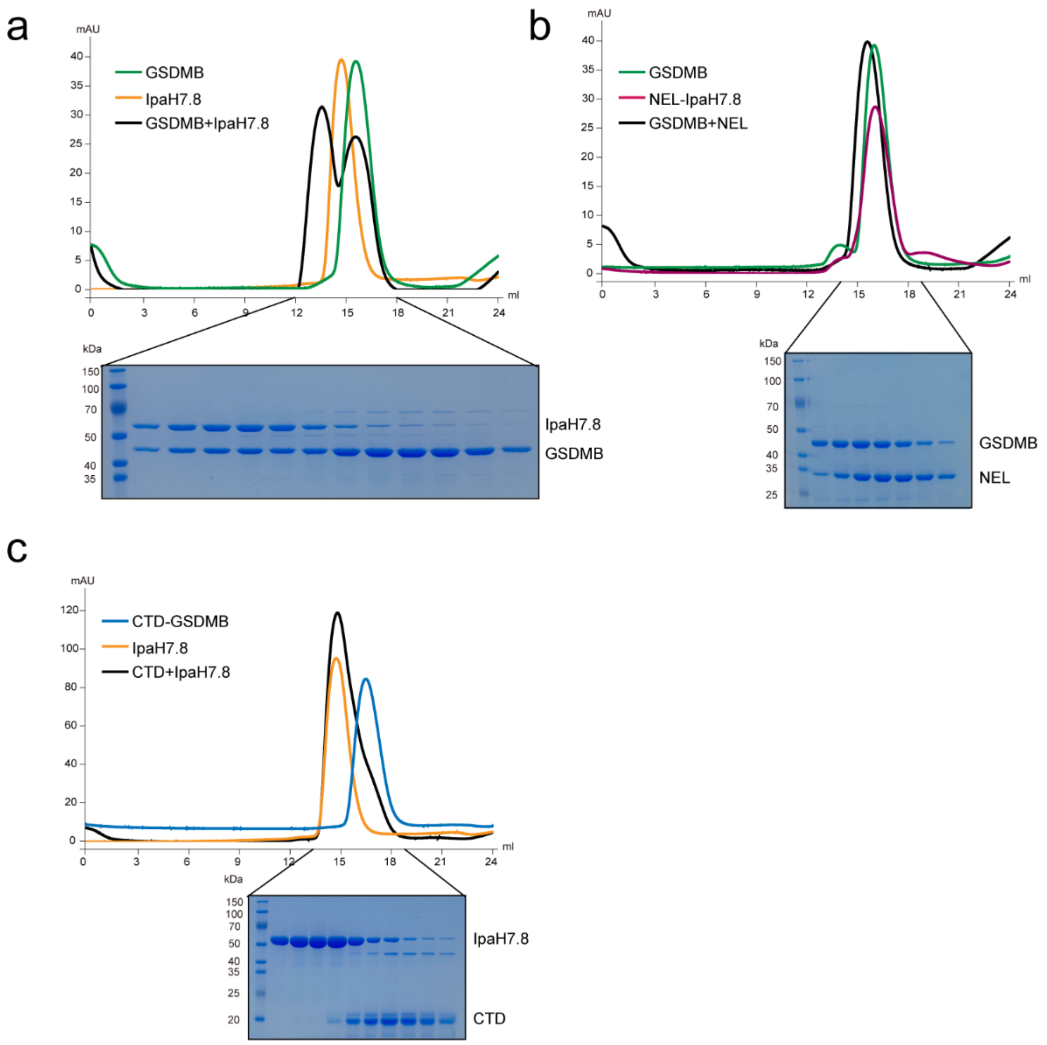

Supplementary Figure 1. The GSDMB-IpaH7.8 association is mediated by NTD-GSDMB and LRR-IpaH7.8 regions. a-b, Overlaid size-exclusion chromatograms of GSDMB and IpaH7.8 (a) or NEL -IpaH7.8 (b). Shown on the bottom are SDS-PAGE analysis of the elution fractions corresponding to GSDMB-IpaH7.8 complex. **c,** Overlaid size-exclusion chromatograms of CTD-GSDMB and IpaH7.8. In Supplementary Fig. 1a–c, experiments were repeated at least twice independently with similar results.

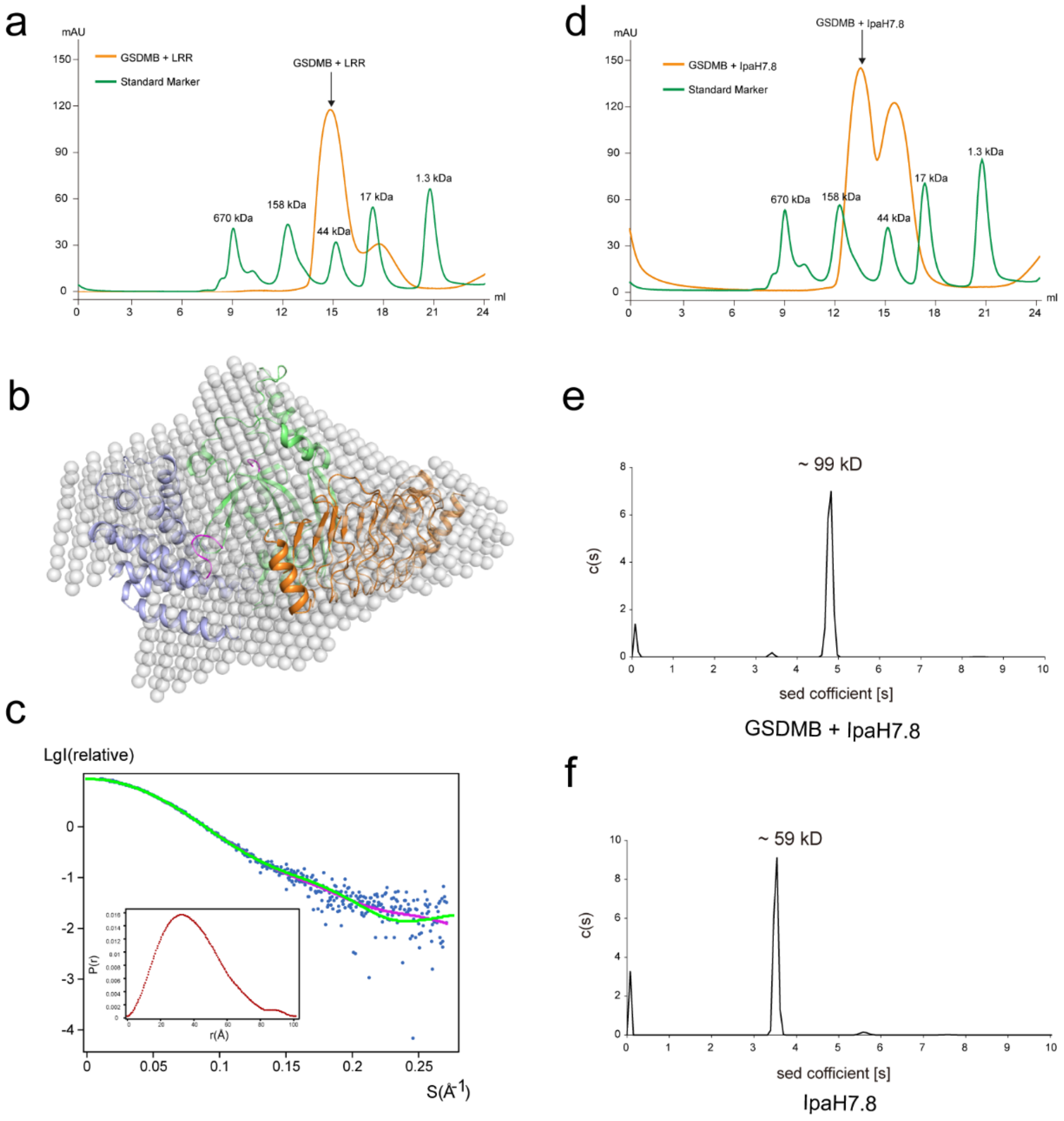

Supplementary Figure 2. IpaH7.8 and GSDMB form a heterodimer in solution. a, Overlaid gel filtration profiles of GSDMB-LRR complex (theoretical heterodimer molecular mass of ~75 kDa) and standard marker. **b,** Superposition of the crystal structure of GSDMB-LRR complex and the ab initio SAXS model (white sphere). **c,** SAXS scattering data of GSDMB-LRR complex (blue dashes) overlaid with the scattering pattern of the ab initio model (green) and the theoretical scattering profile of GSDMB-LRR crystal structure (pink). Inset: SAXS P(*r*) distance distributions of the GSDMB-LRR complex calculated by GNOM. **d,** Overlaid gel filtration profiles of GSDMB-IpaH7.8 complex (theoretical heterodimer molecular mass of ~109 kDa) and standard marker. **e-f,** Sedimentation coefficient distributions of GSDMB-IpaH7.8 complex (e) or IpaH7.8 alone (f). The GSDMB-IpaH7.8 complex has a measured mass of ~99 kDa compared with the theoretical heterodimer molecular mass of ~109 kDa. For apo IpaH7.8 (residues 23-565), the theoretical molecular mass of ~62 kDa compared with its experimental monomeric molecular mass of ~59 kDa.

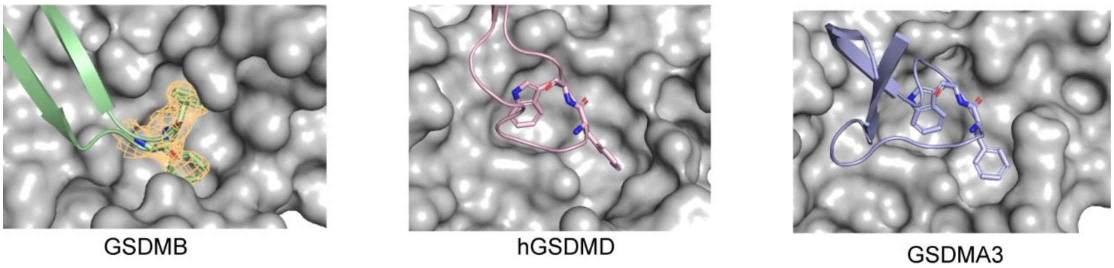

Supplementary Figure 3. Structural comparisons of gasdermins. The hydrophobic pockets from CTD are involved in binding of NTD. The CTDs are shown in surface representations. The 2Fo-Fc electron density map of the residues F46 and F47 in GSDMB contoured at 1.0 σ is colored in orange; GSDMD, PDB code: 6N9O; GSDMA3, PDB code: 5B5R.

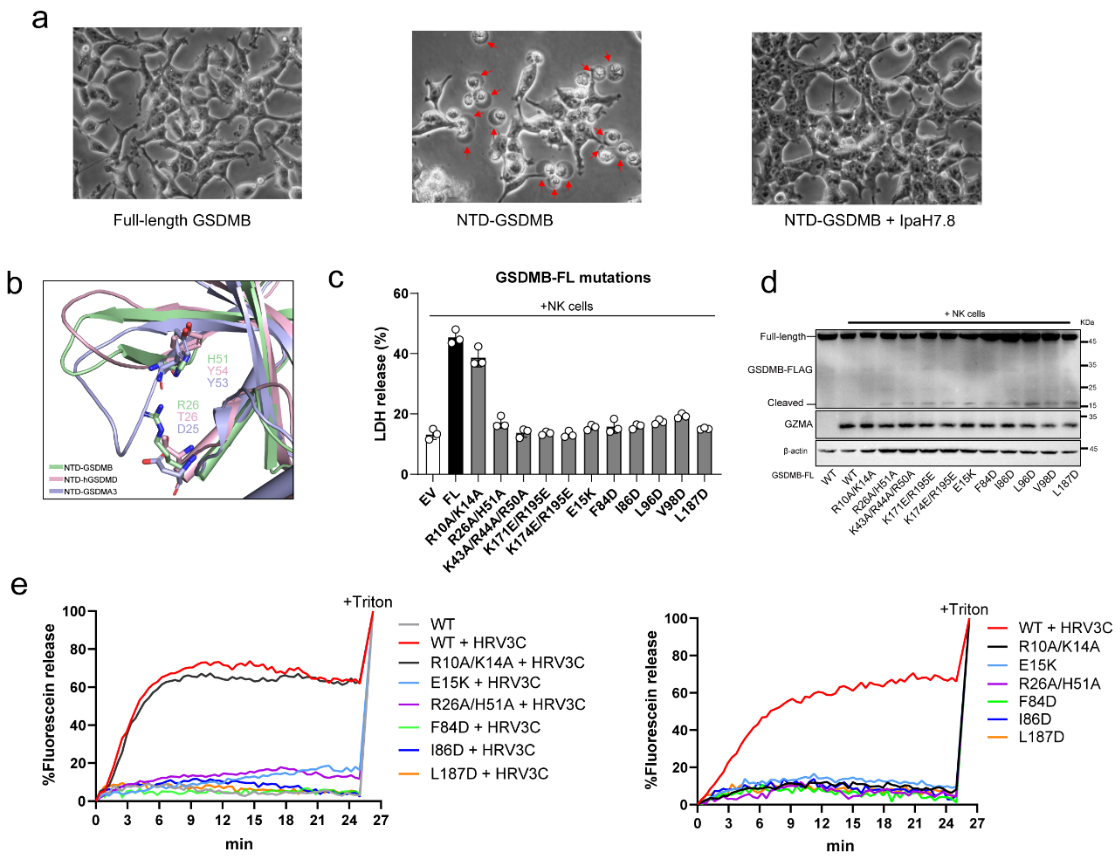

Supplementary Figure 4. NTD of GSDMB is responsible for pore formation. a, Effects of the pore-formation and pyroptosis with indicated proteins in HEK293T cells. Red arrows indicate pyroptotic cells. **b,** Structural comparison of GSDMB (green) with human GSDMD (hGSDMD) (pink) and GSDMA3 (light blue). The unique basic patch consisting of Arg26 and His51 in GSDMB is shown as sticks. **c,** NK cells-mediated cell death was measured by LDH release in GSDMB-expressing HEK293T cells. All experiments were repeated at least three times (mean ± sd, n=3 independent biological replicates). **d,** Expression of GSDMB and GZMA. The figure is related to Supplementary Fig. 4c. **e,** Effects of GSDMB mutations on the liposome leakage. The liposome leakage activities were assessed by measuring the dye release. Data are representative of three independent experiments.

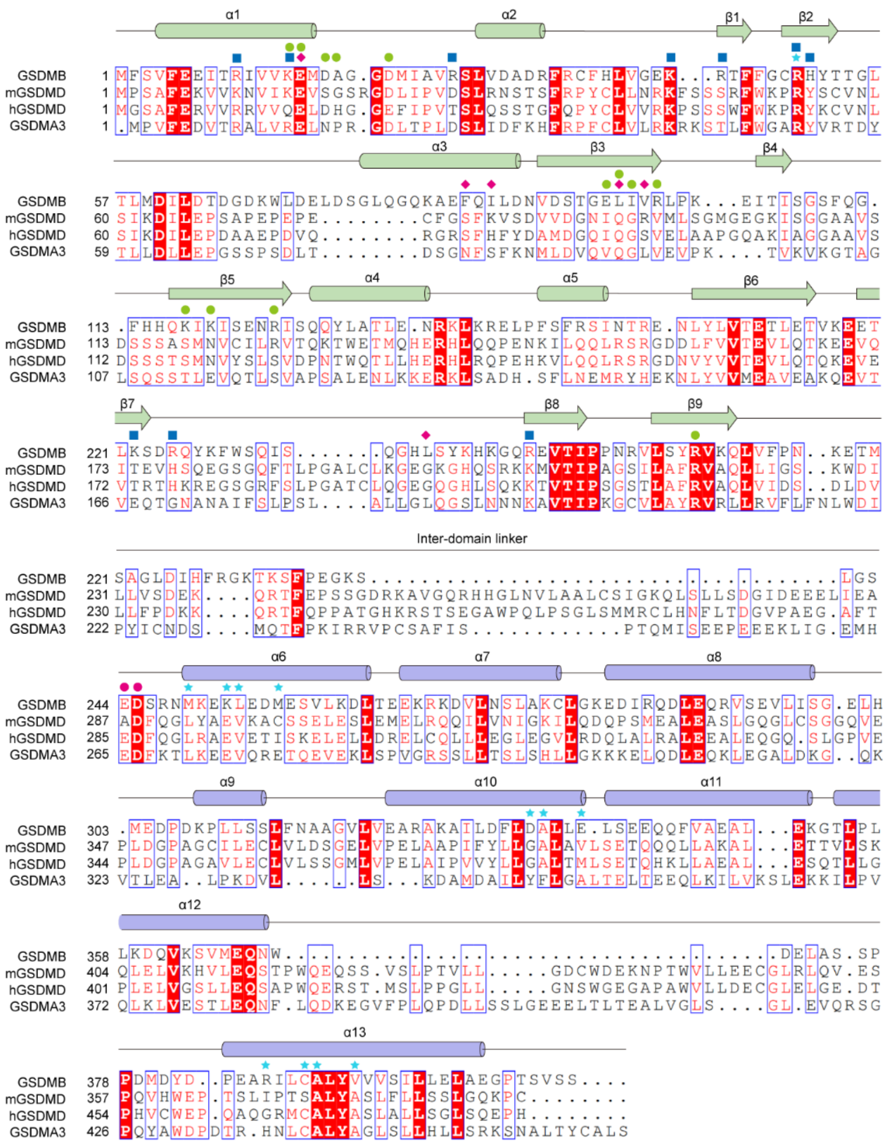

Supplementary Figure 5. Sequence alignment of GSDMB, GSDMD and GSDMA3. Secondary structural elements of GSDMB are indicated above the sequences. The residues crucial for auto-inhibition are indicated by cyan stars. The residues of the inter-domain linker involved in regulation of auto-inhibition are indicated by pink dots. The residues mediating interactions between GSDMB and IpaH7.8 are indicated by green dots. The potential lipid recognition and oligomerization sites are indicated by blue cubes and pink diamonds, respectively. hGSDMD: human GSDMD; mGSDMD: mouse GSDMD.

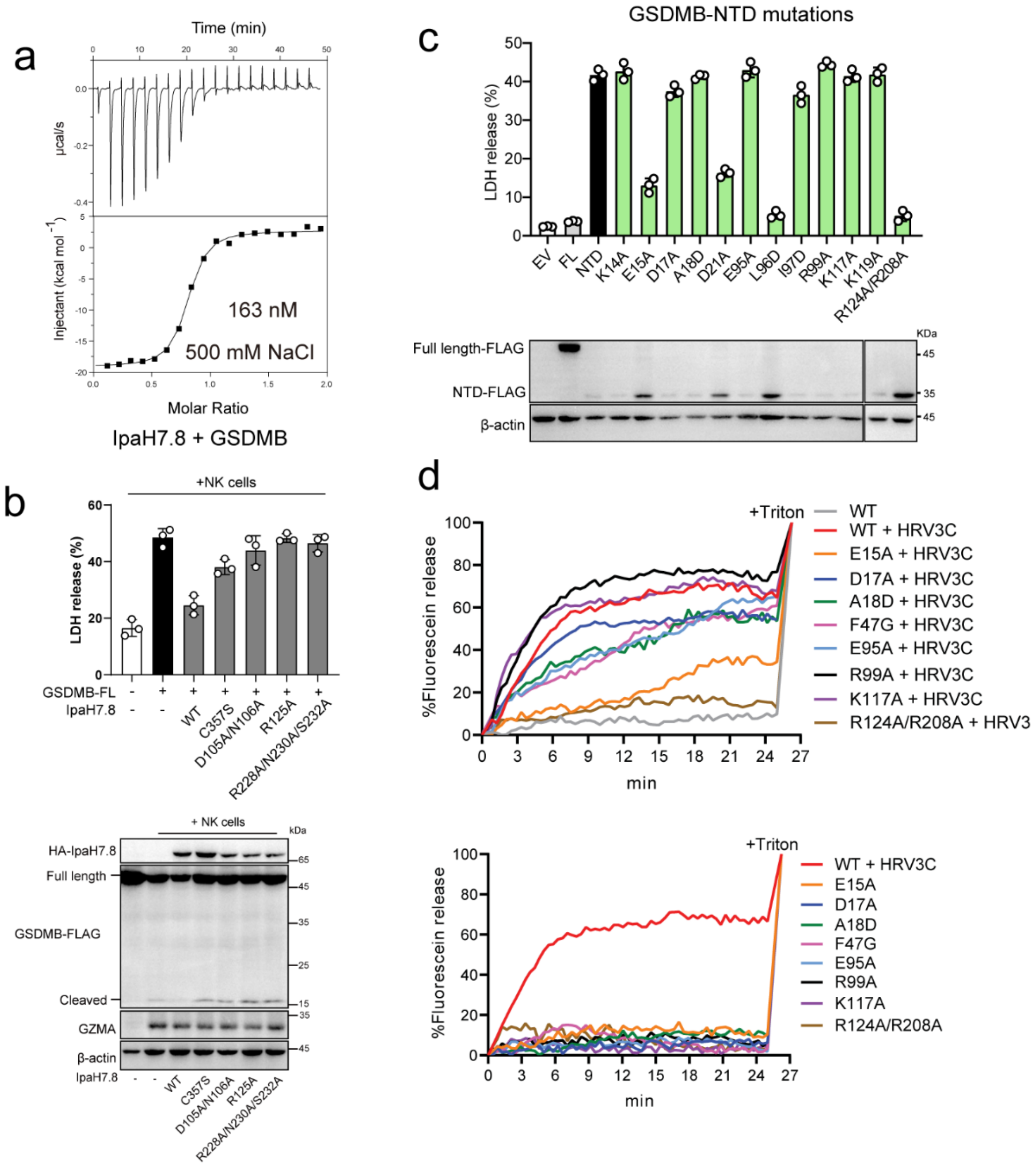

Supplementary Figure 6. Biochemical and functional characterizations of the interaction between GSDMB and IpaH7.8. a, ITC titration curves of IpaH7.8 to GSDMB in a buffer containing 500 mM NaCl. **b,** NK cells-mediated cell death was measured by LDH release in GSDMB-expressing HEK293T cells in the presence of IpaH7.8. All experiments were repeated at least three times (mean ± sd, n=3 independent biological replicates). **c,** Effects of the GSDMB mutations involved in binding of IpaH7.8 on NTD-mediated cell death. The data shown are representative of at least three independent experiments (mean ± sd, n = 3 independent biological replicates). Expression of GSDMB is shown at the bottom. **d,** Effects of GSDMB mutations on the liposome leakage. Data are representative of three independent experiments.

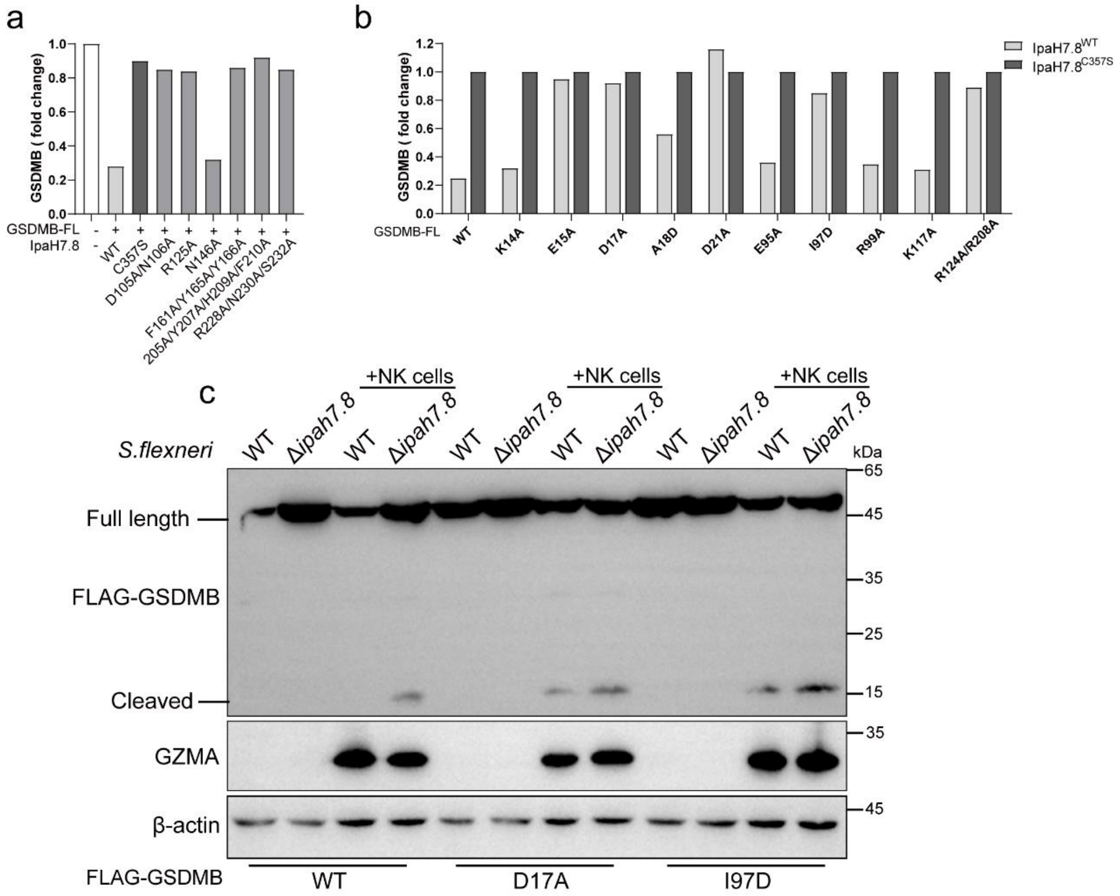

Supplementary Figure 7. The protein expression levels. a-b, The ratios of GSDMB to β-actin are plotted. The two figures (a-b) are related to Fig. 5c and 5f, respectively. **c,** The expression levels of GSDMB and GZMA proteins. Three independent experiments were repeated with similar results. The figure is related to Fig. 5g.

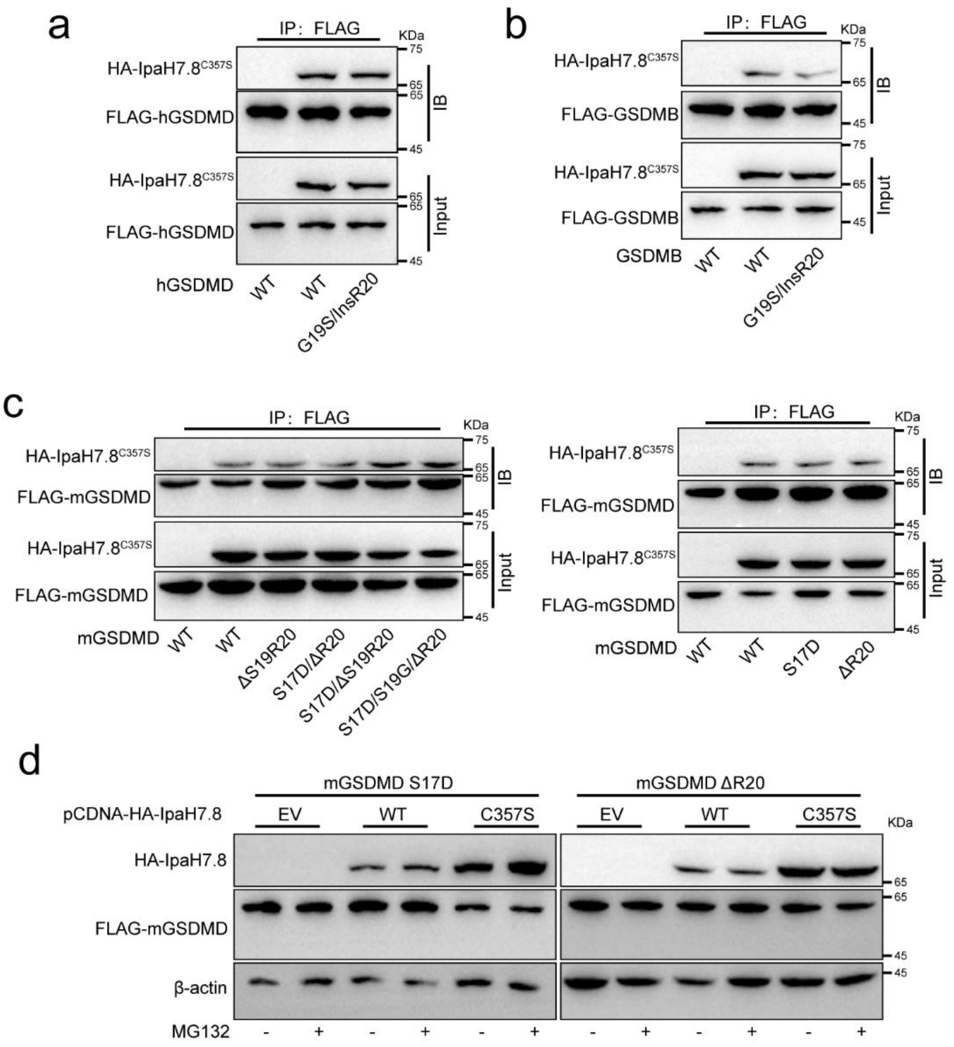

Supplementary Figure 8. IpaH7.8 interacts with GSDMD and GSDMB. a, Human GSDMD (hGSDMD) co-immunoprecipitated with IpaH7.8. FLAG-hGSDMD or empty vector (EV) was co-transfected with HA-IpaH7.8 in HEK293T cells. **b,** GSDMB co-immunoprecipitated with IpaH7.8. FLAG-GSDMB or empty vector (EV) was co-transfected with HA-IpaH7.8 in HEK293T cells. **c,** Mouse GSDMD (mGSDMD) co-immunoprecipitated with IpaH7.8. FLAG-mGSDMD or empty vector (EV) was co-transfected with HA-IpaH7.8 in HEK293T cells. **d,** Cellular degradation assays for mouse GSDMD (mGSDMD). In Supplementary Fig. 8a–d, experiments were repeated at least three times independently with similar results.

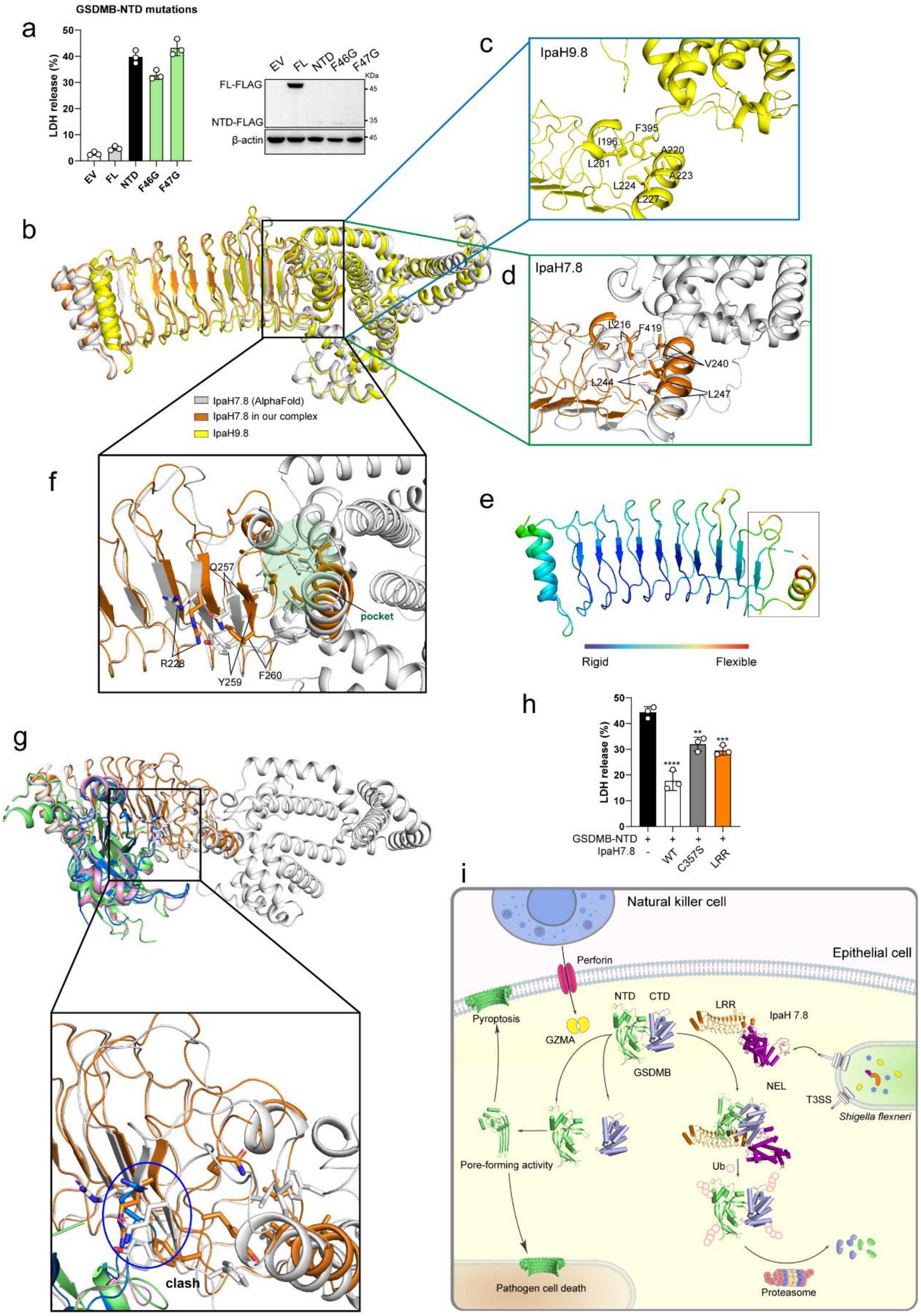

Supplementary Figure 9. IpaH7.8-mediated ubiquitination of GSDMB. a, Mutations of the Phe46 and Phe47 in GSDMB have little effect on LDH release (mean ± sd, n = 3 independent biological replicates). **b,** Structural comparison of the LRR domains in IpaH9.8 (PDB: 6LOL) and IpaH7.8 (AlphaFold2) with that of IpaH7.8 in GSDMB-IpaH7.8 complex. **c-d,** Close-up view of the hydrophobic pocket present in

the LRR C-terminus in IpaH9.8 (c) and IpaH7.8 (d). **e,** The C-terminus of LRR of IpaH7.8 in GSDMB-LRR complex is flexible. **f,** Structural alignment showed that the residues in the C-terminus of LRR undergo large movement upon GSDMB binding. The hydrophobic pocket is highlighted by green. **g,** structural analysis revealed that the α1-α2 loop of mGSDMD would coincide spatially with the C-terminus of LRR of IpaH7.8. **h,** The NTD-GSDMB was co-transfected with the IpaH7.8 plasmids for the LDH assay (mean ± sd, n = 3 independent biological replicates). Statistical differences were calculated using One-way ANOVA as GSDMB-NTD+ IpaH7.8- group as control. (**p = 0.0022, ***p = 0.0004, ****p < 0.0001). **i,** The proposed model of GSDMB-mediated pyroptosis and bacterial lysis, and IpaH7.8-mediated degradation of GSDMB.

Uncropped scans of blots and gels

Supplementary Fig. 1

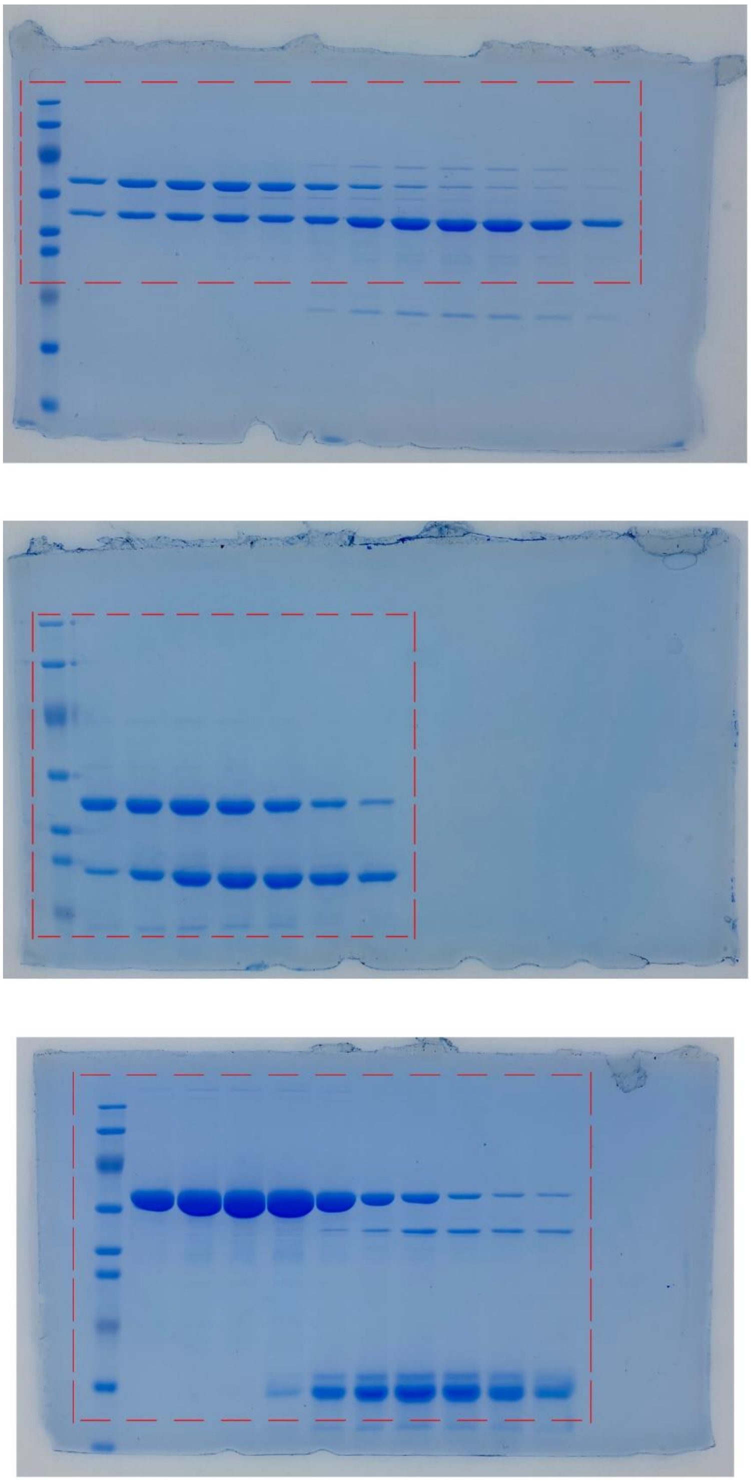

Supplementary Fig. 4d

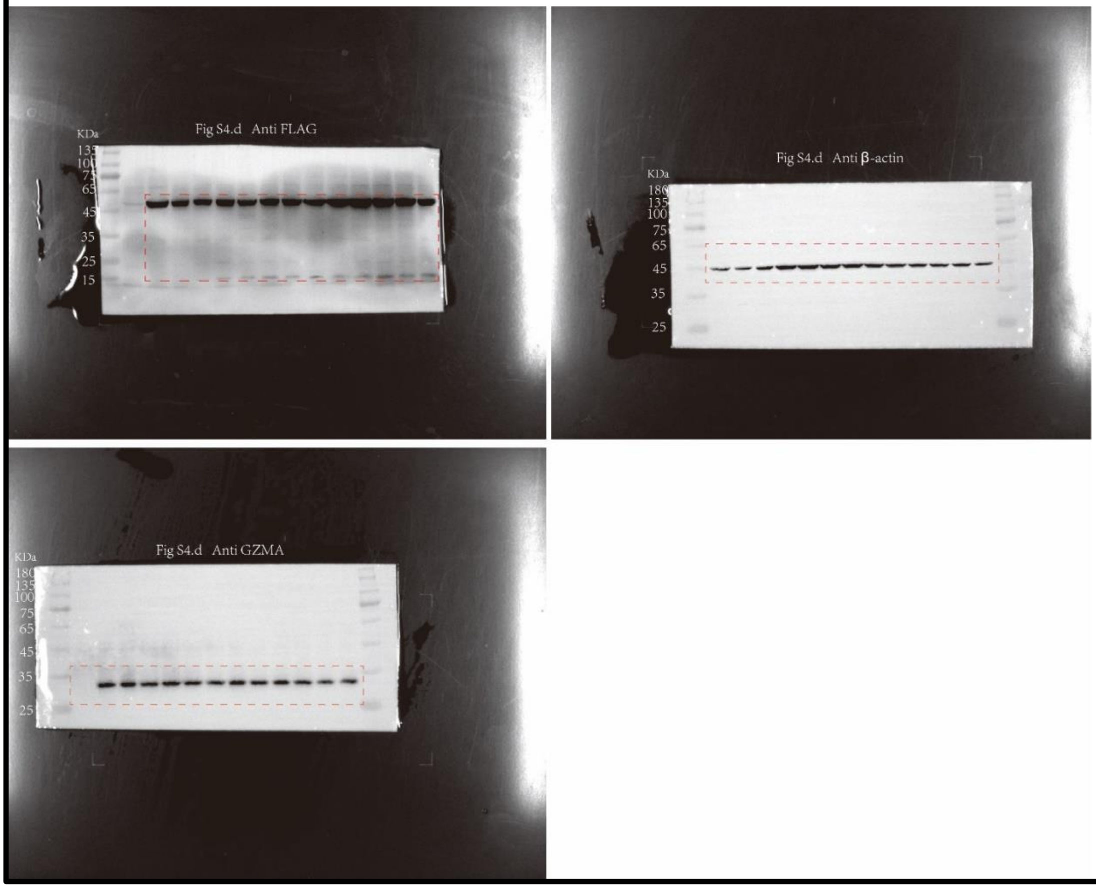

Supplementary Fig. 6b

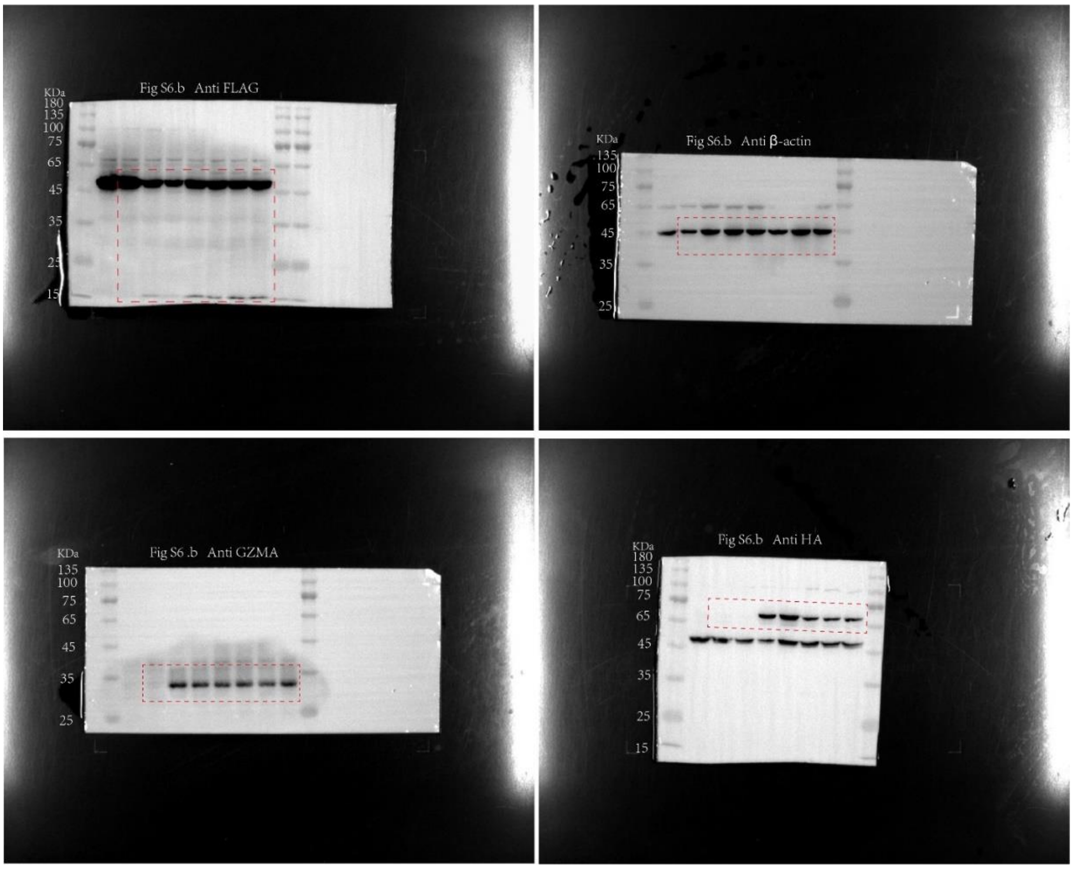

Supplementary Fig. 6c

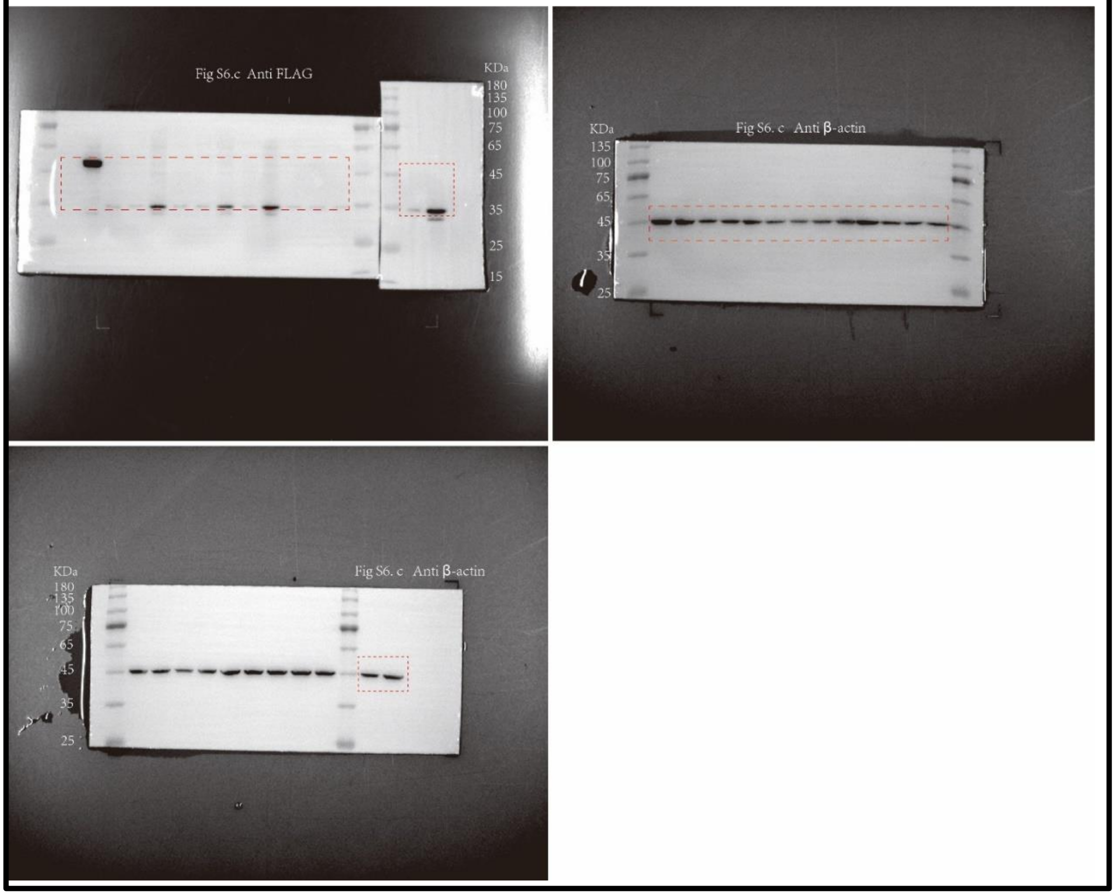

Supplementary Fig. 7c

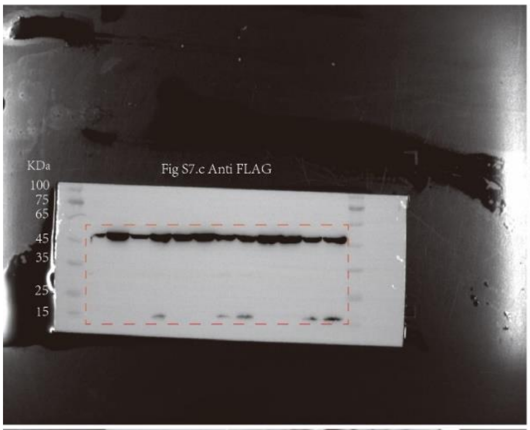

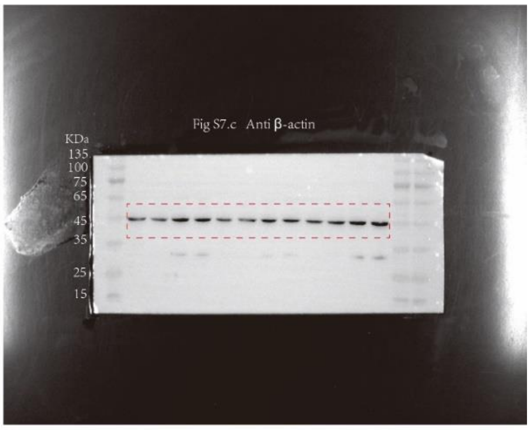

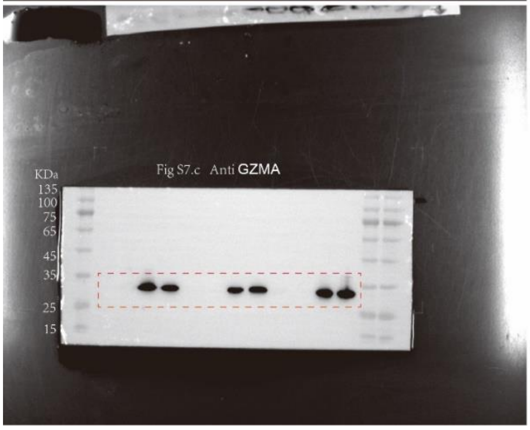

Supplementary Figs. 8a-c

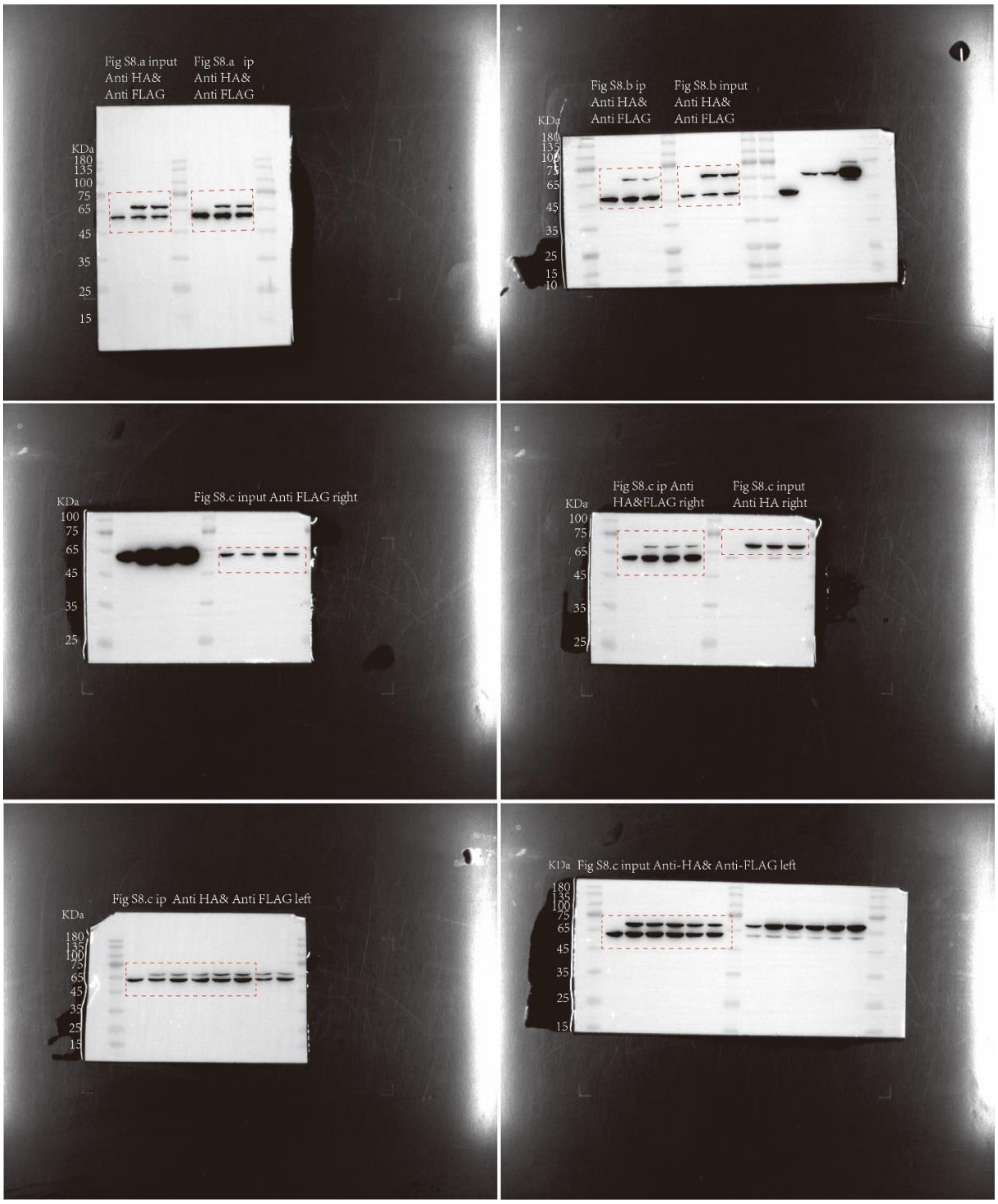

Supplementary Fig. 8d

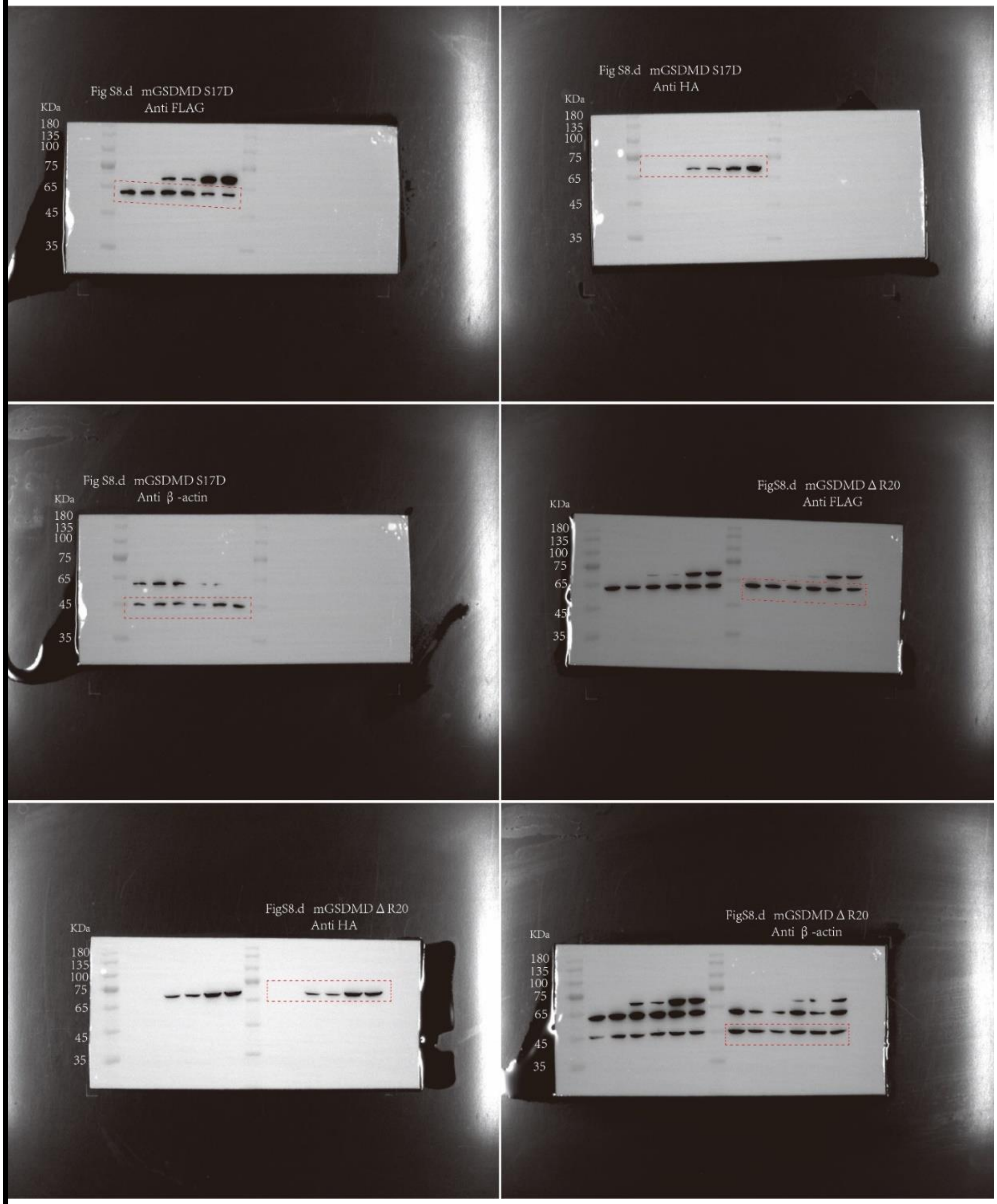

Supplementary Fig. 9a

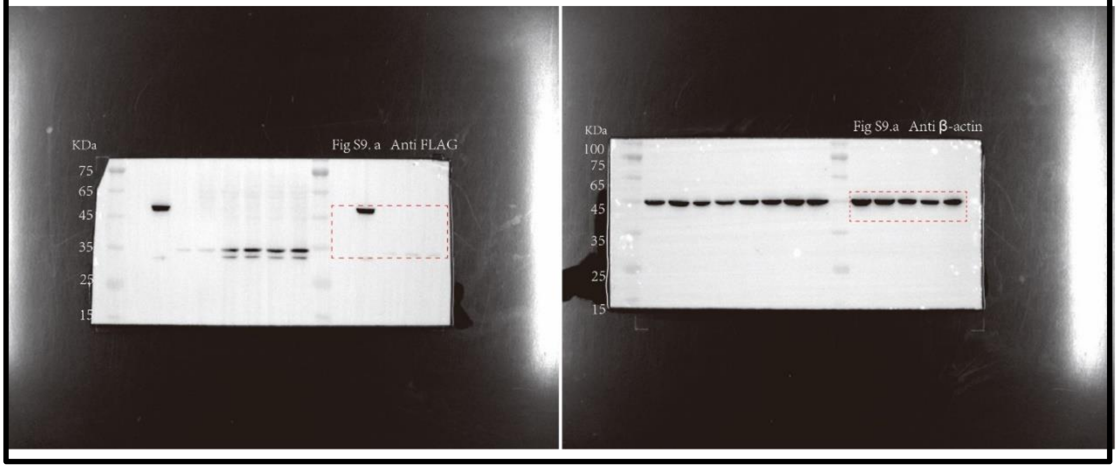

Supplement: Supplementary file 1 — Supplementary Information [file 41467_2022_35725_MOESM1_ESM.pdf]
