# Supplementary material for: Insights into the GSDMB-mediated cellular lysis and its targeting by IpaH7.8
Source: Nat Commun. 2023 Jan 4;14:61. doi: 10.1038/s41467-022-35725-0 (PMC9813358; doi:10.1038/s41467-022-35725-0)
Supplement: Supplementary file 2 — Reporting Summary [file 41467_2022_35725_MOESM2_ESM.pdf]

nature portfolio

Corresponding author(s): Heng Zhang

Last updated by author(s): Dec 7, 2022

Reporting Summary

Nature Portfolio wishes to improve the reproducibility of the work that we publish. This form provides structure for consistency and transparency in reporting. For further information on Nature Portfolio policies, see our Editorial Policies and the Editorial Policy Checklist.

Statistics

For all statistical analyses, confirm that the following items are present in the figure legend, table legend, main text, or Methods section.

n/a	Confirmed	
☐	☒	The exact sample size (*n*) for each experimental group/condition, given as a discrete number and unit of measurement
☒	☐	A statement on whether measurements were taken from distinct samples or whether the same sample was measured repeatedly
☐	☒	The statistical test(s) used AND whether they are one- or two-sided<br>*Only common tests should be described solely by name; describe more complex techniques in the Methods section.*
☒	☐	A description of all covariates tested
☒	☐	A description of any assumptions or corrections, such as tests of normality and adjustment for multiple comparisons
☐	☒	A full description of the statistical parameters including central tendency (e.g. means) or other basic estimates (e.g. regression coefficient) AND variation (e.g. standard deviation) or associated estimates of uncertainty (e.g. confidence intervals)
☐	☒	For null hypothesis testing, the test statistic (e.g. *F*, *t*, *r*) with confidence intervals, effect sizes, degrees of freedom and *P* value noted<br>*Give P values as exact values whenever suitable.*
☒	☐	For Bayesian analysis, information on the choice of priors and Markov chain Monte Carlo settings
☒	☐	For hierarchical and complex designs, identification of the appropriate level for tests and full reporting of outcomes
☒	☐	Estimates of effect sizes (e.g. Cohen's *d*, Pearson's *r*), indicating how they were calculated

Our web collection on statistics for biologists contains articles on many of the points above.

Software and code

Policy information about availability of computer code

Data collection	X-ray diffraction data were collected at beamline BL18U1 of Shanghai Synchrotron Radiation Facility (SSRF) using a Pilatus3 6M detector.
Data analysis	All diffraction data sets were automatically processed using autoPROC. Structure was solved by molecular replacement with CCP4i2 (version 1.1.0). Automated model building was performed by PHENIX (version 1.20-4487) and CCP4i2(version 1.1.0). Improvement of the initial model was carried out manually by COOT (version 0.9.8) , and the refinement was conducted using PHENIX (version 1.20-4487) and REFMAC5 of CCP4 (version 8.0).

For manuscripts utilizing custom algorithms or software that are central to the research but not yet described in published literature, software must be made available to editors and reviewers. We strongly encourage code deposition in a community repository (e.g. GitHub). See the Nature Portfolio guidelines for submitting code & software for further information.

Data

Policy information about availability of data

All manuscripts must include a data availability statement. This statement should provide the following information, where applicable:

- Accession codes, unique identifiers, or web links for publicly available datasets
- A description of any restrictions on data availability
- For clinical datasets or third party data, please ensure that the statement adheres to our policy

The structures in the manuscript has been deposited at the Protein Data Bank (PDB) under the PDB accession code 7WJQ. The CTD of GSDMB (PDB ID:5TJ4) was used as a searching model for molecular replacement to solved the structure of IpaH7.8-GSDMB complex.

Field-specific reporting

Please select the one below that is the best fit for your research. If you are not sure, read the appropriate sections before making your selection.

☒ Life sciences ☐ Behavioural & social sciences ☐ Ecological, evolutionary & environmental sciences

For a reference copy of the document with all sections, see nature.com/documents/nr-reporting-summary-flat.pdf

Life sciences study design

All studies must disclose on these points even when the disclosure is negative.

Sample size	All biological and cell biological experiments used in this study were repeated at least three times.
Data exclusions	No data were excluded in this study.
Replication	All the experiments in this work were repeated at least three times. We detailed the replication of all the experiments in figure legends and method session in the manuscript.
Randomization	Our study focused on the function of proteins only through structural biology, biochemistry and cell biology approaches, and no animal or human subject was used in this work, so randomization is not applicable for the all experiments mentioned in the manuscript.
Blinding	We performed all the experiments using automated methods, so blinding is not applicable for this study.

Reporting for specific materials, systems and methods

We require information from authors about some types of materials, experimental systems and methods used in many studies. Here, indicate whether each material, system or method listed is relevant to your study. If you are not sure if a list item applies to your research, read the appropriate section before selecting a response.

Materials & experimental systems

n/a	Involved in the study	
☐	☒	Antibodies
☐	☒	Eukaryotic cell lines
☒	☐	Palaeontology and archaeology
☒	☐	Animals and other organisms
☒	☐	Human research participants
☒	☐	Clinical data
☒	☐	Dual use research of concern

Methods

n/a	Involved in the study	
☒	☐	ChIP-seq
☒	☐	Flow cytometry
☒	☐	MRI-based neuroimaging

Antibodies

Antibodies used

Anti HA (CST, 3724S, clone: C29F4) Dilution 1:2000
https://www.cellsignal.cn/products/primary-antibodies/ha-tag-c29f4-rabbit-mab/3724?site-search-type=Products&N=4294956287&Ntt=ha&fromPage=plp

Anti FLAG (CST, 14793S, clone: D6W5B) Dilution 1:2000
https://www.cellsignal.cn/products/primary-antibodies/dykddddk-tag-d6w5b-rabbit-mab-binds-to-same-epitope-as-sigma-s-anti-flag-m2-antibody/14793?_=1670414436110&Ntt=FLAG&tahead=true

Anti GZMA (Abcam, ab209205, clone: EPR20161) Dilution 1:1000
https://www.abcam.cn/granzyme-a-antibody-epr20161-ab209205.html#lb

Anti β-actin (Proteintech, 20536-1-AP) Dilution 1:1000
https://www.ptgcn.com/products/ACTB-Antibody-20536-1-AP.htm

HRP linked Anti-Rabbit IgG antibody (CST, 7074S) Dilution 1:10000
https://www.cellsignal.cn/products/secondary-antibodies/anti-rabbit-igg-hrp-linked-antibody/7074?site-search-type=Products&N=4294956287&Ntt=secondary+antibody&fromPage=plp

Validation

High quality commercial monoclonal antibodies were used for biological and cell biological experiments in this study. References for all the monoclonal antibodies can be obtained from the manufacturer's website listed above.

Eukaryotic cell lines

Policy information about cell lines

Cell line source(s)	HEK293T cells (ATCC), NK92MI cells (ATCC).
Authentication	HEK293T and NK92MI cell lines mentioned in the manuscript were from ATCC, so no additional authentication was performed in this study.
Mycoplasma contamination	Cell lines used in this work were routinely tested for mycoplasma every month, and all cell lines were tested negative for mycoplasma contamination.
Commonly misidentified lines (See ICLAC register)	No commonly misidentified cell lines were used in our study.
